# Evaluating the Impact of Orthostatic Syncope and Presyncope on Quality of Life: A Systematic Review and Meta-Analysis

**DOI:** 10.3389/fcvm.2022.834879

**Published:** 2022-02-10

**Authors:** Brooke C. D. Hockin, Natalie D. Heeney, David G. T. Whitehurst, Victoria E. Claydon

**Affiliations:** ^1^Department of Biomedical Physiology and Kinesiology, Simon Fraser University, Burnaby, BC, Canada; ^2^International Collaboration on Repair and Discoveries (ICORD), University of British Columbia, Vancouver, BC, Canada; ^3^Faculty of Health Sciences, Simon Fraser University, Burnaby, BC, Canada

**Keywords:** vasovagal syncope, postural orthostatic tachycardia syndrome (POTS), orthostatic hypotension, carotid sinus hypersensitivity, quality of life

## Abstract

**Purpose:**

Syncope (transient loss of consciousness and postural tone) and presyncope are common manifestations of autonomic dysfunction that are usually triggered by orthostasis. The global impact of syncope on quality of life (QoL) is unclear. In this systematic review, we report evidence on the impact of syncope and presyncope on QoL and QoL domains, identify key factors influencing QoL in patients with syncopal disorders, and combine available data to compare QoL between syncopal disorders and to population normative data.

**Methods:**

A comprehensive literature search of academic databases (MEDLINE (PubMed), Web of Science, CINAHL, PsycINFO, and Embase) was conducted (February 2021) to identify peer-reviewed publications that evaluated the impact of vasovagal syncope (VVS), postural orthostatic tachycardia syndrome (POTS), or orthostatic hypotension (OH) on QoL. Two team members independently screened records for inclusion and extracted data relevant to the study objectives.

**Results:**

From 12,258 unique records identified by the search, 36 studies met the inclusion criteria (VVS: *n* = 20; POTS: *n* = 13; VVS and POTS: *n* = 1; OH: *n* = 2); 12 distinct QoL instruments were used. Comparisons of QoL scores between patients with syncope/presyncope and a control group were performed in 16 studies; significant QoL impairments in patients with syncope/presyncope were observed in all studies. Increased syncopal event frequency, increased autonomic symptom severity, and the presence of mental health disorders and/or comorbidities were associated with lower QoL scores.

**Conclusion:**

This review synthesizes the negative impact of syncope/presyncope on QoL and identifies research priorities to reduce the burden of these debilitating disorders and improve patient QoL.

## Introduction

Orthostatic syncope (fainting; transient loss of consciousness and postural tone) and presyncope (near-fainting) are common manifestations of autonomic dysfunction that are usually triggered by orthostasis (1). The lifetime cumulative incidence of orthostatic syncope is estimated to be between 20 and 40% in the general population (2), and while syncope and presyncope are prevalent across all ages, incidence peaks during adolescence and in older adults (2–4). Although most etiologies of orthostatic syncope carry a benign prognosis, up to one-third of affected individuals experience recurrent and severe episodes (5), and a multitude of studies have reported that syncope is associated with significant impairments in quality of life (6–10).

While orthostatic syncope is a heterogeneous condition, it typically results when autonomic cardiovascular compensation for the gravitational fluid shifts that occur in the upright posture are inadequate, compromising homeostatic blood pressure control and culminating in hypotension and cerebral hypoperfusion (1). The most common syncopal/presyncopal disorders are vasovagal syncope (VVS), postural orthostatic tachycardia syndrome (POTS), orthostatic hypotension (OH) and carotid sinus hypersensitivity (CSH) (11).

The most prevalent form of orthostatic syncope is VVS, accounting for ~60% of total cases and ~80% in pediatric populations (12, 13). In patients with VVS, cardiovascular adaptive mechanisms generally compensate for the upright posture for several minutes before abruptly reversing, leading to widespread vasodilation that may or may not be accompanied by bradycardia (14). The resulting hypotension and cerebral hypoperfusion are often associated with intensifying presyncopal symptoms (light-headedness, nausea, pallor, sweating, tunnel-vision, palpitations, etc.) that ultimately culminate in syncope (2).

POTS is the second most common orthostatic disorder in children and young adults, characterized by chronic (>6 months) and debilitating orthostatic presyncopal symptoms and excessive orthostatic heart rate increases in the absence of hypotension (15).

Responsible for ~15% of orthostatic syncope cases, OH refers to an abnormal decrease in blood pressure upon assuming the upright posture (falling more than 20/10 mmHg within 3 mins of standing), predominantly affecting older adults, and patients with diabetes, hypertension or neurodegenerative disease. OH occurs where cardiovascular adaptive mechanisms are unable to compensate for orthostatic stress; this can be a reflection of a structural (e.g., neuropathy or denervation) or functional (e.g., prescription medications) disruption of autonomic reflexes, known as autonomic failure (16).

CSH is reported to underlie ~30% of unexplained orthostatic syncope in the elderly (17). It is particularly common in older males, with a higher incidence in those with cardiovascular disease or neurodegenerative disorders (18). The precise mechanism of CSH is unclear, but it presents with profound hypotension and bradycardia with mechanical or orthostatic stimulation of the carotid baroreceptors that culminates in presyncope and/or syncope (19).

The impact of chronic orthostatic syncope and presyncope on daily life can be devastating. Presyncopal symptoms can be severe and unrelenting; individuals may suffer fall-related injuries, and recurrent events may cause fear and distress, and lead to decreased school attendance or community engagement, job loss and loss of independence (6). The diagnostic process for patients is often long and stressful, with 35% of patients seeing 10-20 physicians before receiving a diagnosis (20) and 10% remaining undiagnosed a year after presenting to clinic (21). Typically, clinical investigations are focussed on ruling out less common (but potentially life-threatening) causes for the events, such as cardiac arrhythmia or structural heart disease (cardiac syncope) (11). Furthermore, the management of these disorders is challenging, and often ineffective at preventing symptoms entirely. Patient counseling and lifestyle advice are considered primary management recommendations, with a focus on reassuring the patient their condition is not life threatening (11). Many studies have shown that quality of life is significantly reduced in patients with orthostatic syncope (6–10). The peak age of onset of VVS and POTS is between 10 and 15 years of age (3, 4); thus, the onset of these quality of life detriments would predominantly occur during adolescence, with the potential for long term impact.

While these studies have shown that quality of life is reduced in patients with orthostatic syncope and presyncope, the global impact of orthostatic syncope on quality of life remains somewhat unclear. Studies have used a wide variety of instruments to quantify quality of life impairments, making it difficult to reconcile their results. It is also unclear which quality of life domains are predominantly affected by orthostatic syncope and presyncope, how quality of life impairments differ between orthostatic syncopal disorders, and what key issues are causing these impacts. To begin to answer these questions and ultimately identify ways to improve quality of life for patients with recurrent orthostatic syncope and presyncope, a comprehensive review of the literature is required. In this systematic review, we (i) collate and report evidence on the impact of orthostatic syncope and presyncope on quality of life, (ii) evaluate the impact of orthostatic syncopal disorders on domains of quality of life, (iii) identify key factors influencing quality of life outcomes in patients with orthostatic syncopal disorders, and (iv) combine data, where available, to compare quality of life between people with orthostatic syncopal disorders and population normative data.

## Methods

### Databases and Search Strategy

A literature search was conducted to identify peer-reviewed articles, published from database inception to February 2021, in the following databases: MEDLINE (Pubmed), Web of Science, Cumulative Index to Nursing and Allied Health Literature (CINAHL), PsycINFO, and the Embase. The search strategy (Appendix A) combined clinical terms describing orthostatic syncopal disorders with terms describing or related to quality of life. All articles identified in database searches were compiled in Mendeley (version 1.19.8).

### Eligibility Criteria and Study Selection

Studies were selected for inclusion using a two-stage approach. In the first stage, titles, and abstracts were screened to identify studies that investigated patients with syncopal disorders and provided an indication that quality of life was evaluated as an outcome measure. The emphasis at this stage was on excluding articles that were clearly unrelated to the research question, reviewers erred on the side of inclusion to ensure no potentially relevant papers were missed. The requirement for studies to be published in a peer-reviewed journal was incorporated at this first stage. Full-text versions of the remaining articles were obtained prior to the application of the second stage. The study population was required to be made up of children and/or adults with a physician diagnosis (including self-reported physician diagnoses) of orthostatic syncope or presyncope, who had experienced at least one episode in the preceding year. Syncope and presyncope were required to have an autonomic etiology, including VVS (also known as neurally-mediated syncope or reflex syncope), POTS, OH (also known as autonomic failure), and CSH (also known as carotid sinus syndrome). Studies were excluded if syncope or presyncope in the primary study population was secondary to arrhythmia, structural heart disease, metabolic disease or epilepsy. Studies were required to have used a generic quality of life instrument as an outcome measure. With regard to study design, case reports, case series and review articles were excluded. Studies were not excluded based on language; translation of relevant information (for extraction) was sought for articles published in languages other than English or French.

In both stages of the screening process, two team members (BCDH and NDH) evaluated articles for inclusion, independently. Disagreements were resolved through discussion or consultation with a third team member (VEC). Reasons for exclusion of full-text articles (i.e., at stage two) were documented.

### Data Extraction

Data extraction was completed, independently, by two team members (BCDH and NDH). Where available (and relevant), the following data were extracted and compiled in a standardized data extraction form (created in Microsoft Excel): lead author, year of publication, country where research was conducted, study design, population of interest, sample size, participant sex, age of participants, duration of illness/symptoms, mean number of lifetime syncopal episodes, quality of life instrument (s) used, modifications to the quality of life instrument (s), quality of life domain and/or summary scores at baseline and follow-up, results of analyses performed on quality of life data, factors influencing quality of life, and the primary and secondary outcomes of the study. In studies where quality of life scores were reported in a figure only, means and standard deviations (where available) were estimated and extracted using WebPlotDigitizer (Version 4.4; Pacifica, California, USA), a publicly available web-based plot digitizing tool.

### Analytic Considerations and Meta-Analysis Methods

To address aims (i) and (ii)—the collation and reporting of evidence regarding the impact of orthostatic syncope and presyncope on quality of life, and the evaluation of the impact of orthostatic syncopal disorders on domains of quality of life—we consolidated the extracted data from individual studies in review tables. We report data as means and standard deviations; where studies reported data as medians (alongside ranges, interquartile ranges and/or confidence intervals), means and standard deviations were calculated or estimated (22, 23). Narrative summaries of the impacts of orthostatic syncope on overall and domain-specific quality of life are provided.

For aim (iii)—the identification of factors influencing quality of life—we report emerging themes and associated sub-analyses (i.e., where similar analyses are performed across multiple studies).

Finally, to address aim (iv), we performed a meta-analysis to facilitate comparisons of quality of life outcomes between people with different orthostatic syncopal disorders and with population normative data, combining available data from studies, with distinct sample populations, that used the same quality of life instruments. Where two or more studies had significant overlap between their patient samples (mostly follow-up studies), the study with the largest sample size was included in the analysis. For instrument domain and summary scores, pooled means and standard deviations were calculated for each orthostatic syncopal disorder, with weighting based on study sample size. These data were compared to published North American population normative data (where available) and across syncopal disorders using a one-way analysis of variance (ANOVA). Where pooled means and standard deviations were compared between only one syncopal disorder and reference data, a *t*-test was used. For data presented as sample proportions or percentages, weighted proportions were calculated based on study sample sizes for each orthostatic syncope disorder and compared with published population normative data and across syncopal disorders using a chi-square test. All population normative data used in the analyses described in this paper were procured from external sources. Statistical analyses and data visualization were completed using SigmaPlot (Version 14 Systat Software, Inc, San Jose, CA, United States). Data are reported as means ± standard deviations, unless otherwise stated. Where applicable, findings were interpreted using a statistical significance level of *p* < 0.05.

## Results

**Figure 1:**
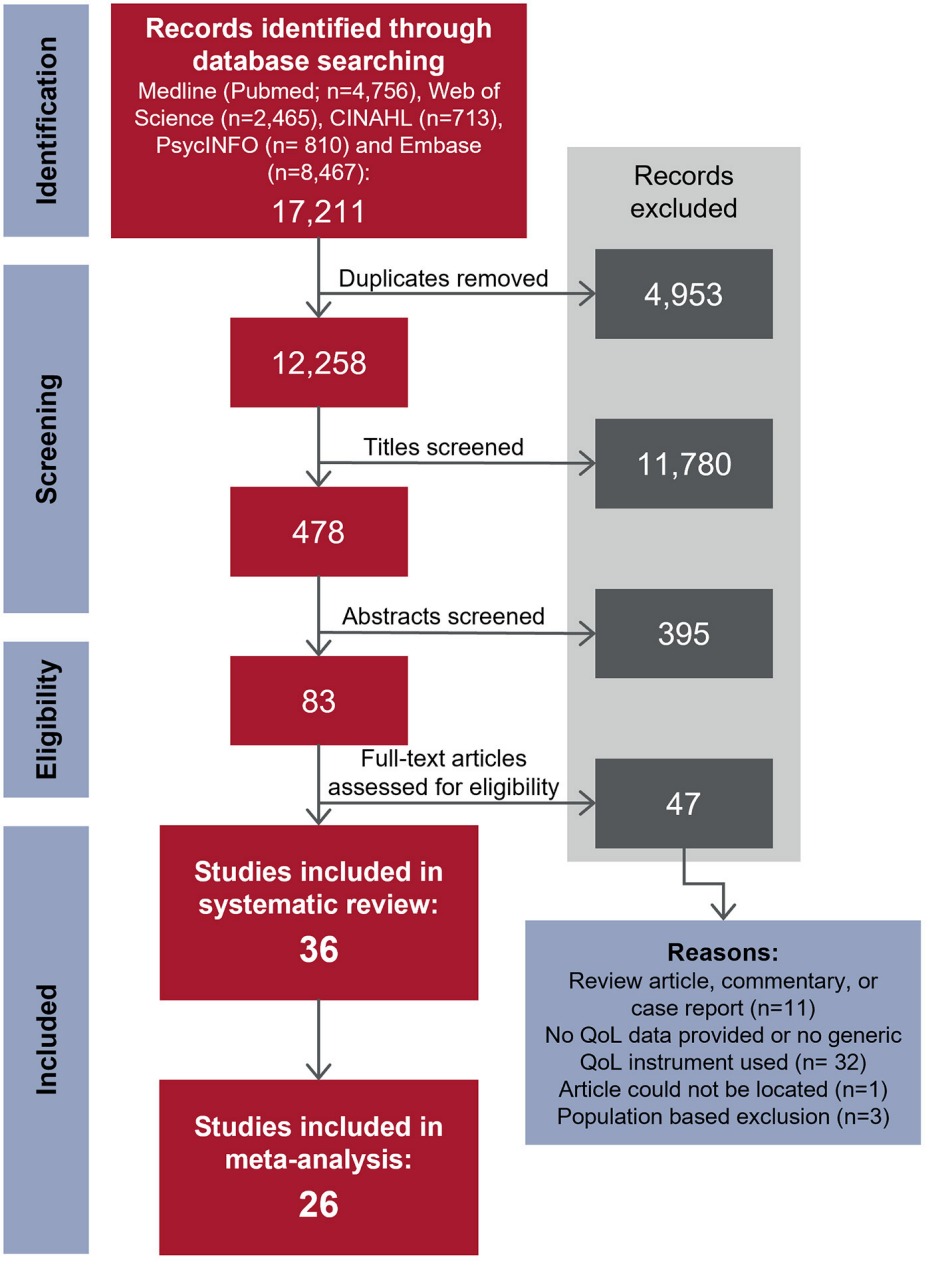
Flowchart depicting the study selection process. Studies were selected for inclusion using a two-stage approach. In stage one (screening), titles, then abstracts were screened to select studies published in peer-reviewed journals that investigated patients with syncopal disorders and provided an indication that quality of life was evaluated as an outcome measure. The emphasis at this stage was on excluding articles that were clearly unrelated to the research question. In stage two (eligibility), full-text papers for stage one inclusions were obtained and inclusion and exclusion criteria were firmly applied. Reasons for exclusion were recorded in the second stage of the study selection process. Adapted from PRISMA statement (2009) (24).

The database search identified 12,258 unique articles; 36 articles met the inclusion criteria. A consort diagram detailing the number of articles included and excluded at each stage, including reasons for exclusion of full-text articles, is shown in Figure 1. Study characteristics of the 36 articles are summarized in Table 1, by population of interest (VVS: *n* = 20; POTS: *n* = 13; VVS and POTS: *n* = 1; OH: *n* = 2; CSH: *n* = 0). In five instances, more than one article reported relevant data from the same or overlapping sample populations [i. (25) and (26); ii. (38) and (39); iii. the first Prevention of Syncope Trial (POST1) (30) and (31); iv. the second Prevention of Syncope Trial (POST2) (10, 30) and (53)].

**Table 1:** Study characteristics.

**Study**	**Country**	**Study design**	**Sample size** **(% females)**	**Age** **(years)**	**Duration of illness/** **symptoms (years)**	**Mean # of lifetime syncopal episodes**	**Quality of life outcome measure used**
**Vasovagal syncope**							
Baron-Esquivias et al. (25)	Spain	Cross-sectional	271 (51)	45 ± 20	3.2 ± 4.9^*^	3.3 ± 2.2^*^	SF-36
Baron-Esquivias et al. (26)	Spain	Longitudinal	167 (57)	44 ± 31^*^	3.3 ± 5.1^*^	3.7 ± 3.0^*^	SF-36
Baron-Esquivias et al. (27)	Spain and Canada	RCT	46 (52)	56 ± 11	–	14 ± 8^*^	SF-36
Van Dijk et al. (28)	Netherlands	Cross-sectional	385 (42)	52 ± 19	3.3 ± 5.8^*^	5 ± 6^*^	SF-36
Van Dijk et al. (21)	Netherlands	Longitudinal	268 (39)	53 ± 18	4.1 ± 7.3^*^	5 ± 7^*^	SF-36
Giada et al. (29)	Italy	Cross-sectional	61 (67)	44 ± 18	18 ± 13^*^	31 ± 22^*^	SF-36
Ng et al. (30)	North America, Germany, Columbia	RCT	280 (32)	39 ± 17	Onset age: 22 ± 17	15 ± 19^*^	SF-36
Sheldon et al. (31)	North America, Germany, Columbia	RCT	204 (65)	42 ± 18	13 ± 16^*^	11 ± 11^*^	SF-36 EQ-5D-3L
Rose et al. (32)	Canada	Cross-sectional	136 (58)	40 ± 17	5.0 ± 11.4^*^	9.5 ± 14.9^†^	EQ-5D-3L
Rose et al. (33)	Canada	Cross-sectional	114 (68)	40 ± 16	9.6 ± 12.2^*^	12 ± 14^*^	EQ-5D-3L
Atici et al. (34)	Turkey	Cross-sectional	88 (61)	35 ± 14	–	9.3 ± 6.0^*^	EQ-5D-3L
Kovalchuk (35)	Ukraine	Case-control	56 (43)	14 ± 2	Onset age: 12.7 ± 2.9	3.4 ± 5.3	PedsQL 4.0
Anderson et al. (8)	USA	Cross-sectional	106 (65)	15 ± 3^*^	94% within 6 months	1: 39%; 1-3: 32%; 3-6: 19%; > 6: 10%	PedsQL 4.0
Capitello et al. (36)	Italy	Cross-sectional	125 (48)	13 ± 3	–	> 1: 73.6% (*n* = 92)	PedsQL 4.0
Ng et al. (10)	North America Germany, Columbia	Case-control	76 (68)	34 ± 14	14 ± 17^*^	24 ± 32^*^	RAND-36 Global health VAS
Santhouse et al. (37)	UK	Case control	52 (NA)	36 ± 15	–	4 (past year)	WHOQOL-BREF
St-Jean et al. (38)	Canada	Cross-sectional	104 (65)	49 ± 17		5 (median)	QLSI
Lévesque et al. (39)	Canada	Longitudinal	73 (66)	50 ± 17	–	5 (median)	QLSI
Broadbent et al. (40)	Australia	Case-control	47 (NA)	49 ± 18	1.3 ± 1.9 since HUT	10.0 ± 15.3 since HUT	PWI-A
Linzer et al. (7)	USA	Cross-sectional	62 (71)	49 ± 19	0.92 (median)	10 (median)	SIP
**Postural orthostatic tachycardia syndrome**							
Bhatia et al. (41)	USA	Cross-sectional	172 (84)	22 ± 2	5.4 ± 1.9 (since diagnosis)	–	SF-36
Benrud-Larson et al. (9)	USA	Cross-sectional	94 (88)	34 ± 10	7.5 ± 5.7	–	SF-36
Benrud-Larson et al. (42)	USA	Cross-sectional	94 (88)	34 ± 10	7.5 ± 5.7	–	SF-36
Rodriguez et al. (43)	Switzerland	Case-control	8 (75)	25 ± 10^*^	–	–	SF-36
Hutt et al. (44)	USA	Cross-sectional	255 (91)	34 ± 11	–	–	SF-36
Moon et al. (45)	Korea	Cross-sectional	107 (63)	31 ± 11	–	–	SF-36
Anderson et al. (46)	Australia	Case-control	15 (80)	30 ± 3	–	–	SF-36
George et al. (47)	USA	Cohort study	251 (86)	26 ± 11	≥6 months	–	SF-36v2
Gibbons et al. (48)	USA	Pragmatic treatment trial	77 (90)	26 ± 6	4.5 ± 1.9 years	–	EQ-5D-3L (EQ VAS only)
Bagai et al. (49)	USA	Case-control	44 (89)	36 ± 11	> 6 months	–	EQ-5D-3L, RAND-36
Fisher et al. (50)	USA	Cross-sectional	58 (91)	37 ± 10	5.8 ± 5.3^*^	–	PROMIS-10
Pederson et al. (51)	USA	Cross-sectional	624 (97)	34 ± 11	10.7 ± 11.9	–	CDC HRQOL-14
Pederson et al. (52)	USA	Cross-sectional	360 (100)	34 ± 12	9.3 ± 9.2	–	WHOQOL-BREF
**Vasovagal syncope and postural orthostatic tachycardia syndrome**							
Hall et al. (53)	North America	Cross sectional	VVS: 72 (67)	34 ± 14	–	≥3	RAND-36
			POTS: 177 (93)	31 ± 11	–	–	
**Orthostatic hypotension**							
Kim et al. (54)	South Korea	Cross-sectional	117 (77)	76 ± 6	–	–	EQ-5D-3L
Francois et al. (55)	USA	Longitudinal treatment trial	115 (NA)	63 ± 17	10 ± 11.4	–	SF-8

*Duration of illness/symptoms refers to the time since first symptoms experienced. In some studies, the duration of the illness was reported as the time since diagnosis, rather than since first symptom presentation. Abbreviations: Randomized Controlled Trial (RCT); head-upright tilt test (HUT)*.

* *Mean and standard deviation estimated from median and interquartile range, or median and range*.

† *Geometric mean reported*.

Across the 36 studies, 12 distinct generic quality of life instruments were used: the Medical Outcomes Study 36-item Short Form Health Survey (SF-36) (*n* = 15); SF-36 version 2 (SF-36v2) (*n* = 1); Medical Outcomes Study 8-item Short Form Health Survey (SF-8) (*n* = 1); the three-level version of the EQ-5D (EQ-5D-3L) (*n* = 7); the Pediatric Quality of Life inventory (PedsQL) version 4.0 (*n* = 3); the RAND 36-item Health Survey (RAND-36) (*n* = 3); the Quality of Life Systemic Inventory (QLSI) (*n* = 2); the Sickness Impact Profile (SIP) (*n* = 1); the World Health Organization Brief Quality of Life Questionnaire (WHOQOL-BREF) (*n* = 2); the Patient-Reported Outcomes Measurement Information System (PROMIS) Global Health (PROMIS-10) (*n* = 1); the Healthy Days Core Module (CDC HRQOL-14) (*n* = 1); and the Personal Wellbeing Index—Adult form (PWI-A) (*n* = 1). One further study reported results for a global health visual analog scale (VAS) where the anchors were identical to the VAS that used to be part of the EQ-5D-3L [i.e., 0 = “Worst imaginable health state” and 100 = “Best imaginable health state”; the wording of the VAS in the EQ-5D-3L has since changed (57)] (10); for the purposes of this review, these data will be placed in context with other studies that used the VAS in the EQ-5D-3L (known as EQ VAS). All quality of life instruments described here, including assessment details, domains, scoring information and normative data used in subsequent analyses, are described in Appendix B.

Individual study results, including baseline quality of life scores, between-group comparisons and additional contextual findings are reported in Table 2. To avoid redundancy in our reporting, data from three longitudinal studies (26, 35, 36) have been omitted from Table 2; their results, including baseline scores, comparisons and contextual findings can be found in Table 3. We note that these three studies report follow-up data from three prior studies that are described in Table 2 (26, 41, 42). Statistical comparisons of quality of life scores between patients with orthostatic syncope or presyncope and a control group or reference population were performed in 15 (42%) studies (8 VVS, 6 POTS, 1 OH). Seven of these studies used the SF-36, two used the RAND-36, four used the EQ-5D-3L index scores and/or EQ VAS, and two used the PedsQL; no other quality of life instrument was used in more than one study.

**Table 2:** Study results.

**Study**	**Outcome measure**	**Baseline scores – population of interest (mean ±SD)**	**Comparisons**	**Additional noteworthy findings**
**Vasovagal syncope**				
Baron-Esquivias et al. (25)	SF-36 ^*^SD estimated from interquartile range; physical and mental component summary scores calculated based on domain scores	Physical functioning: 80.5 ± 22 Role physical: 62.5 ± 75 Bodily pain: 66.6 ± 43 General health: 60.9 ± 24 Vitality: 59.8 ± 26 Social functioning: 77.6 ± 28 Role emotional: 62.6 ± 75 Mental health: 64.4 ± 26.8 **Physical component summary:** 47 ± 13 **Mental component summary:** 45 ± 16	All SF-36 domain scores except role physical and role emotional were lower in the VVS group compared to Spanish normative data (p-values not reported). Compared to reference data from patients with heart failure, VVS patients scored similarly in 5 SF-36 domains; patients with heart failure scored worse in general health, vitality and physical functioning domains (*p* ≤ 0.009). Within the VVS group, women scored lower in all SF-36 domains compared to men (*p* < 0.05). When comparisons to Spanish norms were stratified by sex, women with VVS scored lower in all SF-36 domains, while men with VVS scored lower in only 4 domains (not specified).	When SF-36 data were stratified by age group (18-24, 25-34, 34-44, 45-54, 55-64, 65-74, > 75 years; 26 patients < 18 years excluded), quality of life in all SF-36 domains was found to decrease with age (*p* < 0.05). These relationships were stronger in women and in women all SF-36 domains decreased with age (*p* < 0.05), while in men this relationship was significant in 6 of 8 domains (*p* < 0.05) (all except role physical and bodily pain). The number of lifetime syncope spells were weakly but significantly (*p* < 0.002) related to all SF-36 domain scores except physical functioning and role physical. Symptom duration was weakly but significantly related to bodily pain, general health and mental health scores. Syncope frequency (# events/symptom duration) and tilt table test result (positive or negative) were not related to quality of life scores.
Baron-Esquivias et al. (27)	SF-36 ^*^mean and SD for role physical, general health, role emotional, and mental health domains, and both summary scores estimated from figures	Physical functioning: 79.2 ± 6.1 Role physical: 50.5 ± 11.9 Bodily pain: 68.2 ± 8.4 General health: 61.1 ± 5.1 Vitality: 54.4 ± 6.7 Social functioning: 70.4 ± 7.4 Role emotional: 60.5 ± 11.2 Mental health: 65.6 ± 6.1 **Physical component summary:** 46.1 ± 2.5 **Mental component summary:** 42.2 ± 3.5	At baseline, the eight subdomain scores were lower in VVS patients compared to Spanish population norms, with greater differences in physical and emotional role limitations (statistics not reported, unsure if these differences were significant).	
Van Dijk et al. (28)	SF-36 ^*^weighted means and SD calculated from male and female data	Physical functioning: 67 ± 28 Role physical: 44 ± 44 Bodily pain: 64 ± 28 General health: 54 ± 24 Vitality: 49 ± 25 Social functioning: 68 ± 28 Role emotional: 61 ± 44 Mental Health: 66 ± 23 **Physical component summary:** 43 ± 10 **Mental component summary:** 43 ± 13	Patients scored significantly lower on all scales (domain and summary scores) of the SF-36 compared to Dutch normative data (*p* < 0.01).	In a univariate model, age, gender, number of episodes in the last year, number of presyncopal episodes and the level of comorbidity (Charlston comorbidity score) were associated with physical component summary scores. These factors were added to a multivariate model, which showed female gender, the presence of presyncopal episodes, and a higher comorbidity were associated with decreased physical component summary scores (*p* < 0.01). A shorter duration of symptoms and the presence of presyncopal episodes were associated with reduced mental component summary scores in both the univariate and multivariate (*p* < 0.03) models.
Giada et al. (29)	SF-36 ^*^physical and mental component summary scores calculated based on domain scores	Physical functioning: 80 ± 22 Role physical: 47 ± 40 Bodily pain: 62 ± 30 General health: 56 ± 23 Vitality: 50 ± 19 Social functioning: 62 ± 25 Role emotional: 51 ± 42 Mental Health: 61 ± 20 **Physical component summary:** 45 ± 10 **Mental component summary:** 41 ± 11	Compared to control group, all SF-36 domain scores were significantly reduced in VVS patients (*p* < 0.05). Physical and mental component summary scores were not evaluated. The prevalence of psychiatric disorders was higher in VVS patients than controls (DSM IV criteria−73% vs. 23%, *p* < 0.001) with a high prevalence of anxiety, mood and somatization disorders. Within the VVS group, SF-36 scale scores were lower in patients with psychiatric disorders than those without (*p* < 0.05). SF-36 domain scores did not differ between patients with and without syncope-related injury, or between those younger or older than 40 years old.	Syncopal recurrence during the follow-up period (mean duration: 15 ± 2 months) was more common in VVS patients with psychiatric disorders (58% vs. 17%; *p* < 0.05). The presence of psychiatric disorders was significantly predictive of syncopal recurrence in the follow-up period (hazard ratio 6.94, 95% CI: 1.7-27.6; *p* = 0.006). Number of syncopal episodes over the previous 6 months was correlated with physical functioning, role physical, bodily pain and general health domain scores. Lifetime syncopal episodes were correlated with physical functioning and role physical domain scores, and general health and physical function domain scores were significantly reduced in patients with ≥ 6 lifetime episodes.
Ng et al. (30)	SF-36	Physical functioning: 78 ± 23 Role physical: 59 ± 40 Bodily pain: 67 ± 25 General health: 64 ± 22 Vitality: 50 ± 22 Social functioning: 71 ± 27 Role emotional: 72 ± 38 Mental health: 67 ± 20 **Physical component summary:** 46 ± 10 **Mental component summary:** 46 ± 11		Quality of life in all SF-36 domains except bodily pain improved in patients with VVS after enrolling in clinical trial, independent of randomization to drug or placebo and independent of syncope recurrence.
Sheldon et al. (31)	SF-36	**Physical component summary:** 48 ± 10 **Mental component summary:** 45 ± 10		
	EQ-5D-3L	**EQ-5D-3L index:** 0.81 ± 0.22 **EQ VAS:** 74 ± 18		
Rose et al. (32)	EQ-5D-3L ^*^no SD provided for EQ VAS scores	*Prevalence (%) of patients reporting any limitations or problems* Mobility: 25.9% Usual activities: 37.2% Self care: 9.0% Pain/discomfort: 49.2% Anxiety/depression: 43.4%	The prevalence of limitations was greater in syncope patients compared to healthy controls in all five dimensions of the EQ-5D-3L. EQ VAS scores were not different between low and high syncope risk groups (*p* = 0.221).	There was a significant curvilinear relationship between EQ VAS scores and the log frequency of syncope spells (*p* < 0.01). When patients were stratified by syncope risk, there was a significant negative linear relationship between EQ VAS scores and the log frequency of syncope spells in the high-risk syncope group (≥6 spells; slope = −5.9; SE 1.1; *p* < 0.001). This was not observed in the low-risk group. The frequency of syncope was higher in patients with impairments in mobility and usual activity domains (*p* ≤ 0.001). Multiple linear regression analysis showed that in high-risk patients four of the EQ-5D-3L dimensions (mobility, usual activities, anxiety/depression and pain/discomfort) and log frequency of syncope spells were independent predictors of EQ VAS scores (*p* ≤ 0.045).
		**EQ VAS:** Low risk syncope group (<6 spells): 72.6 (*n* = 59) High risk syncope group (≥6 spells): 68.5 (*n* = 74)		The total number of comorbidities also tended to be associated with decreased VAS scores in this group (*p* = 0.057). In the low-risk group, pain, impaired mobility and level of comorbidity were significant predictors of EQ VAS scores (*p* ≤ 0.036). There was no significant effect of age, gender or tilt test outcome in either regression model.
Rose et al. (33)	EQ-5D-3L	**EQ VAS:** 70.6 ± 10.9		-
Atici et al. (34)	EQ-5D-3L	**EQ-5D-3L index:** 0.48 ± 0.22 **EQ VAS:** 73.6 ± 14.3	No differences in EQ-5D-3L index or EQ VAS scores were found between VVS VASIS subtypes.	The EQ-5D-3L index (r = −0.649, *p* < 0.001) and EQ VAS (r = −0.587, *p* < 0.001) were significantly and negatively correlated with total syncope episodes and were statistically important parameters related to total syncope episodes in a stepwise multiple linear regression analysis (*p* < 0.030).
Kovalchuk (35)	PedsQL 4.0	*Self-report score; proxy-report score* **Total score:** 67.92 ± 14.52; 65.13 ± 13.94 Physical health: 72.27 ± 15.44; 67.18 ± 16.58 Psychosocial health: 65.54 ± 15.52; 64.06 ± 15.47 Emotional functioning: 60.18 ± 19.42; 60.71 ± 18.13 Social functioning: 72.97 ± 19.86; 71.96 ± 19.74 School functioning: 63.61 ± 16.50; 60.46 ± 17.18	Child self-report and parent proxy-report total PedsQL scores and psychosocial health, emotional functioning and social functioning domain scores were reduced in the VVS cohort compared to healthy controls (*p* < 0.0472). Parent proxy report scores in the VVS sample were additionally reduced in the physical health domain compared to healthy controls (*p* = 0.0180)	
Anderson et al. (8)	PedsQL 4.0	*Self-report score; proxy-report score* **Total score:** 75.2 ± 13.9; 73.3 ± 16.7 Physical health: 78.8 ± 14.9; 77.6 ± 18.5 Psychosocial health: 73.9 ± 19.9; 73.1 ± 20.4 Emotional functioning: 68.9 ± 20.7; 67.7 ± 22.3 Social functioning: 86.2 ± 17.1; 80.7 ± 20.8 School functioning: 66.4 ± 22.1; 66.1 ± 24.1	Compared to data from healthy controls, patients with VVS scored significantly lower in physical, psychosocial, emotional and school domains (*p* < 0.001) of the PedsQL and had lower total scores (*p* < 0.0001). Social functioning domain scores were not different. Compared to patients with diabetes mellitus, VVS patients had lower total scores (*p* < 0.0001), and lower physical (*p* < 0.0001), psychosocial (*p* < 0.05) and school (*p* < 0.001) domain scores. Total PedsQL scores were similar to those with asthma, end-stage renal disease, obesity, and structural heart disease. Patients with VVS had better physical health compared to patients with end-stage renal disease (*p* < 0.05), better emotional functioning than patients with asthma or end-stage renal disease (*p* < 0.05), and better social functioning than patients with asthma, obesity, renal disease, or structural heart disease (*p* < 0.0001). School functioning was worse in VVS patients than in obese individuals (*p* < 0.0001).	Proxy-reported social functioning domain scores were reduced compared to child self-reports (*p* < 0.004); proxy- and self- reported scores were otherwise similar. No patient or clinical variables were associated with PedsQL scores.
Capitello et al. (36)	PedsQL 4.0	*Self-report score; proxy-report score* **Total score:** 74.71 ± 14.68; 75.89 ± 16.4 Physical health: 73.38 ± 18.29; 77.22 ± 18.33 Psychosocial health: 75.08 ± 15.76; 75.46 ± 17.99	Children and adolescents displayed significant agreement with parents in terms of how they perceive their overall QoL; levels of agreement increased with age for physical health, but decreased with age for psychosocial well-being. Parent scores were systematically higher than child self-reports, but were not significantly or meaningfully different.	There were no significant correlations between child or parent PedsQL scores and sex, age, recurrent syncope or psychological factors (Child Behavior Checklist: externalizing and internalizing problem scores). Child self-reported psychosocial and total quality of life scores were, however, significantly related to indices of parent stress (Parent Stress Index: parent-child dysfunctional interaction scale, difficult child scale, total parent stress scale).
Ng et al. (10)	RAND-36	Physical functioning: 77 ± 24 Role physical: 33 ± 42 Bodily pain: 68 ± 25 General health: 60 ± 22 Emotional well-being: 69 ± 21 Energy/fatigue: 50 ± 22 Social functioning: 71 ± 24 Role emotional: 54 ± 45 **Physical health composite:** 53 ± 9 **Mental health composite:** 56 ± 11	RAND-36 domain and summary scores and global health VAS scores were all significantly lower in VVS patients compared to healthy controls (*p* < 0.001). More patients with VVS met criteria for “borderline” or “probable” anxiety (*p* < 0.001), “borderline” or “probable” depression (*p* = 0.013; hospital anxiety and depression scale – HADS), and clinically significant anxiety sensitivity (*p* < 0.001) compared to healthy controls.	In patients with VVS there were weak, but significant, negative correlations between anxiety and all RAND-36 dimensions except physical functioning and both summary scores (*p* ≤ 0.026). Depression (HADS) was negatively correlated with all RAND-36 dimensions and summary scores (*p* ≤ 0.002). Anxiety sensitivity (anxiety sensitivity index) was negatively correlated with all RAND-36 dimensions except pain and both summary scores (*p* ≤ 0.01). In healthy controls the only statistically significant relationships were between anxiety and pain, depression and role limitations due to emotional health, and anxiety sensitivity and emotional well-being (*p* ≤ 0.031).
		**Global health VAS:** 71 ± 19		
Santhouse et al. (37)	WHOQOL-BREF	Physical health: 62.7 ± 28.2 Environmental: 64.7 ± 19.1 Psychological: 60.7 ± 23.6 Social relationships: 70.7 ± 19.1 **Overall quality of life:** 4.0 ± 1.5 Quality of health: 4.3 ± 0.8	Overall quality of life (*p* = 0.0001), quality of health (*p* < 0.0001) and physical (*p* < 0.0001), environmental (*p* = 0.0002), and psychological (*p* = 0.002) domain scores were significantly reduced in VVS patients compared to healthy controls. There were no differences between patients with VVS and patients with epilepsy.	The number of syncopal episodes was not correlated with scores on any of the QoL scales.
St-Jean et al. (38)	QLSI	**Global QoL:** 6.35 ± 5.09	Participants were clustered based on their scores on the Illness Representations survey. QLSI scores were increased in those with low perceived illness severity compared to those with intermediate or high perceived illness severity. QLSI scores were comparable between intermediate and high perceived illness severity groups.	There was no significant effect of sex on quality of life, but there was a significant interaction between sex and syncope type. Men with unexplained syncope had lower QLSI scores compared to men with VVS (*p* = 0.004), but not compared to women with unexplained syncope or VVS. Increasing age (*p* = 0.036), more lifetime syncope episodes (*p* = 0.005) and the presence of anxiety or depression disorders (*p* < 0.001) were associated with decreased quality of life.
Broadbent et al. (40)	PWI-A	General life satisfaction (GLS): 69.57 ± 22.65 Health satisfaction (Hsat): 60.64 ± 24.44 Non-health related subjective well-being (PWI-H): 73.62 ± 17.54	Hsat scores were significantly reduced in VVS (*p* = 0.01) and patients with coronary artery disease (CAD) (*p* = 0.004) compared to healthy controls, but were not different between VVS and CAD patients. GLS and PWI-H scores were similar between groups.	Quality of life variables (GLS, Hsat, PWI-H) were negatively correlated with measures of anxiety and depression in all three groups, but these relationships were stronger in the two patient groups than in controls.
Linzer et al. (7)	SIP	**SIP total score:** 16.8 ± 14.2 SIP psychosocial score: 19.9 (SD not reported) SIP physical score: 11.1 (SD not reported)	In syncope patients the SIP psychosocial scores were significantly higher than physical scores (*p* < 0.0001), reflecting greater psychosocial impairment. Total SIP scores in syncope patients were higher than in the general population and comparable to patients with severe rheumatoid arthritis and chronic low back pain (statistics not reported).	SIP physical dimension scores were moderately correlated with age (r = 0.25; *p* < 0.05) and number of co-morbid conditions (r = 0.43; *p* < 0.002). Symptom checklist 90 scores (SCL-90-R, a measure of current point-in-time psychological symptom status) were elevated in patients with VVS compared to reference control data. SIP psychosocial scores were correlated (r > 0.4; *p* < 0.001) with all 9 subscales (somatization, obsessive -compulsive, interpersonal sensitivity, depression, anxiety, hostility, phobic anxiety, paranoia, and psychoticism) of the symptom checklist 90.
**Postural orthostatic tachycardia syndrome**				
Bhatia et al. (41)	SF-36 ^*^33 patients reported complete resolution of POTS symptoms. SF-36 scores do not include these patients (*n* = 139; 84% female)	**Physical component summary:** 33 ± 15.2 **Mental component summary:** 49.4 ± 11.5	Physical component summary scores were significantly decreased compared to population normative data (*p* < 0.001). Mental component summary scores were in the normal range.	The following symptoms were each independently associated (*p* ≤ 0.004) with reductions in SF-36 age- and sex-adjusted physical component summary scores: fatigue, pain, nausea, sleep disturbance, brain fog, memory problems, exercise intolerance, purplish/bluish discoloration of hands and feet, shortness of breath, palpitations, vomiting, dizziness/light-headedness, syncope.
Benrud-Larson et al. (9)	SF-36 ^*^means for SF-36 domains estimated from figures; SD was not shown or reported; physical and mental component summary scores calculated based on domain scores	Physical functioning: 54.2 ± 27.4 [reported for this sample in (38)] Role physical: 33 Bodily pain: 60 General health: 48 Vitality: 36 Social functioning: 59 Role emotional: 81 Mental Health: 72 **Physical component summary:** 34 **Mental component summary:** 49	SF-36 scores in physical functioning, role physical, bodily pain, general health, vitality, and social functioning domains were all significantly reduced compared to reference data for healthy controls (*p* < 0.01), but were similar to reference data for patients with congestive heart failure and chronic obstructive pulmonary disease. Role emotional and mental health scores were similar between the 4 groups. (No comparisons reported for physical or mental component summary scores). A large proportion (24%) of patients reported their employment status as disabled (unable to work due to POTS symptoms); these patients reported decreased scores in physical functioning, role physical, bodily pain, general health, vitality, social functioning, and role emotional domains, and reduced physical component summary scores (*p* < 0.05).	Autonomic Symptom Profile (ASP) scales were more strongly correlated with SF-36 physical component summary (r = −0.49; *p* < 0.01) than mental component summary (r = −0.27; *p* < 0.05) scores. All autonomic symptom scales (upper gastrointestinal symptoms, secretomotor dysfunction, pupillomotor symptoms, vasomotor symptoms, constipation, bladder dysfunction, sleep dysfunction) except diarrhea were significantly correlated (*p* < 0.05) with SF-36 physical component summary scores, but the orthostatic intolerance scale had the strongest correlation (r = −0.45; *p* < 0.01). Sleep dysfunction, upper gastrointestinal and pupillomotor symptoms were the only ASP scales significantly related to the mental component summary score (*p* < 0.05). Disability status and symptom severity were independently associated with physical component summary scores.
Benrud-Larson et al. (42)	SF-36	Physical functioning domain: 54.2 ± 27.4		SF-36 physical functioning domain scores were significantly (*p* < 0.05) correlated with perceived disability (r = −0.78), orthostatic symptom severity (r = −0.46), catastrophizing (r = −0.33), depression symptoms (r = −0.26) and somatic vigilance (r = −0.23).
Rodriguez et al. (43)	SF-36	**Physical component summary:** 43.41 ± 7.29 **Mental component summary:** 46.42 ± 10.58	The physical component summary score was significantly reduced relative to the control group (*p* = 0.003); the mental component summary score was not.	
Hutt et al. (44)	SF-36	**Physical component summary:** 30.5 ± 9 **Mental component summary:** 44.6 ± 10		A large proportion (44%) of POTS patients had abnormally low functional capacity for their age and sex; this was more common in younger patients (*p* = 0.0017). Low functional capacity in POTS patients was associated with reduced physical component summary scores on the SF-36 (*p* = 0.006).
Moon et al. (45)	SF-36	**Physical component summary:** 43.4 ± 8.3 **Mental component summary:** 39.9 ± 11.4		Increased symptoms of orthostatic intolerance were significantly associated with lower physical (r = −0.534) and mental (r = −0.436) component summary scores (*p* < 0.01). All items of the orthostatic intolerance questionnaire (nausea, tremor in hands, dizziness, palpitation, headache, profuse perspiration, blurred vision, chest discomfort, lightheadedness, and concentration difficulties) were correlated with the physical component summary score except tremor in hands, and all were correlated with mental component summary scores except tremor in hands and profuse sweating. The magnitude of the orthostatic heart rate increase was not related to any of the orthostatic intolerance symptoms, or SF-36 physical or mental component summary scores.
Anderson et al. (46)	SF-36	Physical functioning: 70 Role physical: 46 Bodily pain: 62 General health: 30 Vitality: 36 Social functioning: 68 Role emotional: 49 Mental Health: 54 **Physical component summary:** 41 ± 7 **Mental component summary:** 39 ± 14	All eight SF-36 domain scores and both physical and mental component summary scores, were significantly reduced in POTS patients compared to healthy controls (*p* < 0.001).	
George et al. (47)	SF-36v2	Physical functioning: 32 ± 9 Role physical: 27 ± 9 Bodily pain: 40 ± 11 General health: 31 ± 8 Vitality: 32 ± 9 Social functioning: 28 ± 11 Role emotional: 41 ± 13 Mental Health: 42 ± 12 **Physical component summary:** 30 ± 9 **Mental component summary:** 40 ± 12		
Gibbons et al. (48)	EQ-5D-3L	**EQ VAS:** 62 ± 13		
Bagai et al. (49)	EQ-5D-3L	*Prevalence (%) of patients reporting some or extreme problems:* Mobility: 60 Usual activities: 95 Self care: 35 Pain/discomfort: 81 Anxiety/depression: 56 **EQ VAS:** 53 ± 17	More participants with POTS reported some or extreme problems in each of the five dimensions (*p* < 0.0001) of the EQ-5D-3L compared to healthy controls. The EQ VAS scores were significantly lower in POTS patients compared to healthy controls (*p* < 0.0001).	
	RAND-36	Physical functioning: 53 ± 17 Role physical: 26 ± 2 Bodily pain: 39 ± 9 General health: 30 ± 9 Emotional well-being: 47 ± 10 Energy/fatigue: 30 ± 7 Social functioning: 29 ± 11 Role emotional: 41 ± 13 **Physical health composite:** 26 ± 9 **Mental health composite:** 43 ± 11	All RAND-36 dimensions and both summary scores were significantly reduced in patients with POTS compared to healthy controls (*p* ≤ 0.009). Quality of life deficits were greater in physical health domains compared to mental health domains. RAND-36 scores were comparable to reference data from patients with rheumatoid arthritis and end-stage renal disease (significance testing not performed).	Physical (R = −0.70; R^2^ = 0.53; *p* < 0.0001) and mental R = 0.58; R^2^ = 0.26; *p* < 0.0001) composite scores were correlated with sleep problems (Medical Outcomes Study Sleep Problems index).
Fisher et al. (50)	PROMIS-10	**PROMIS-10 mental health T-score:** 37.9 ± 7.0 **PROMIS-10 physical health T-score:** 34.3 ± 6.2		PROMIS-10 physical (r = −0.60; 95% CI:−0.76,−0.38) and mental (r = −0.45; 95%CI:−0.64,−0.15) health T-scores were significantly (*p* < 0.05) correlated (spearman) with autonomic symptoms (from COMPASS-31). There were no significant relationships between time since diagnosis, time since symptoms onset and PROMIS-10 scores.
Pederson et al. (51)	CDC HRQOL-14 (One additional item added assessing number of days with brain fog.)	Days of poor physical health: 19.59 ± 8.81 Days of poor mental health: 13.86 ± 10.14 Days with activity limitations: 17.48 ± 10.06 Days with pain: 15.52 ± 10.95 Days of sadness: 12.17 ± 10.04 Days of worrying: 15.69 ± 11.19 Days without enough sleep or rest: 22.37 ± 9.29 Days with good energy: 2.26 ± 4.07	POTS patients had fewer days with good energy and more days of poor physical and mental health, activity limitations, pain, sadness, worrying, brain fog and without enough sleep or rest compared to healthy controls (*p* < 0.001). More POTS patients rated their health as poor and reported activity limitations and needing help with routine needs and personal care compared to healthy controls (*p* < 0.001).	
		Days with brain fog: 19.34 ± 9.97 (added item) 35.4% rated their health as poor 97.1% reported their activities were limited by illness 76.1% reported needing help with routine needs (e.g., shopping, chores, or conducting business) 30.1% reported needing help with personal care (e.g., eating, bathing, dressing, or getting around the house)		
Pederson et al. (52)	WHOQOL-BREF (Environmental domain excluded to reduce survey length and quality of health question not reported.)	Physical health: 36.0 ± 16.5 Psychological: 41.2 ± 17.4 Social relationships: 46.1 ± 22.0 **Overall quality of life:** 4.9 ± 1.6		A large proportion of participants reported poor (score < 60) physical health (89.2%), psychological health (82.2%) and social relationships (75.0%).
**Vasovagal syncope and postural orthostatic tachycardia syndrome**				
Hall et al. (53)	RAND-36 ^*^VVS group: *n* = 71 for emotional well-being, mental health composite scores ^*^POTS group: *n* = 176 for physical functioning, emotional well-being, role emotional; *n* = 175 for mental health composite; *n* = 174 for general health composite	*VVS scores; POTS scores* Physical functioning: 76.5 ± 24.6; 42.5 ± 22.6 Role physical: 33.0 ± 42.4; 11.4 ± 25.3 Bodily pain: 67.7 ± 24.6; 48.8 ± 25.3 General health: 60.5 ± 22.1; 31.2 ± 20.0 Emotional well-being: 68.9 ± 20.4; 67.4 ± 17.3 Energy/fatigue: 50.7 ± 22.1; 27.2 ± 17.3 Social functioning: 71.2 ± 24.6; 45.2 ± 23.9 Role emotional: 55.6 ± 44.1; 65.7 ± 42.6	RAND-36 scores were reduced in POTS patients compared to VVS in physical functioning, role physical, energy and fatigue, social functioning, pain and general health domains (*p* < 0.001); emotional well-being (*p* = 0.271) and role emotional (*p* = 0.052) domains were not significantly different. The physical health and general health composite scores were lower in POTS compared to VVS (*p* ≤ 0.030), but the mental health composite score was lower in VVS patients than in POTS patients (*p* = 0.005).	When RAND-36 data were stratified by sex (36M; 249F) rather than diagnosis, females reported significantly lower scores in all domains except emotional well-being and role emotional. Physical health composite scores were lower in females, while there was no effect of sex on mental and general health composite scores. Within the VVS group (24M; 48F), male patients reported higher RAND-36 scores in physical functioning (*p* = 0.014), role physical (*p* = 0.005), social functioning (*p* = 0.002) and pain (*p* = 0.040) domains and on physical (*p* = 0.005), mental (*p* = 0.027) and general health composite scores (*p* = 0.015). Within POTS (12M; 177F) patients, males scored lower than females in the energy/fatigue domain and on mental (*p* = 0.038) and general health composite scores (*p* = 0.036).
		**Physical health composite:** 53.1 ± 9.3; 38.1 ± 20.0 **Mental health composite:** 56.1 ± 11.0; 62.4 ± 20.0 **General health composite:** 61.5 ± 11.0; 57.5 ± 18.6		
**Orthostatic hypotension**				
Kim et al. (54)	EQ-5D-3L	Prevalence (%) of patients reporting some or extreme problems: Mobility: 53.8 Usual activities: 28.2 Self care: 16.2 Pain/discomfort: 67.5 Anxiety/depression: 45.3 **EQ-5D-3L index:** 0.56 ± 0.29 **EQ VAS:** 65.79 ± 20.54	More participants in the OH group reported some or extreme problems in each of the five dimensions (*p* ≤ 0.022) of the EQ-5D-3L compared to participants without OH. The EQ-5D-3L index (*p* < 0.001) and EQ VAS (*p* = 0.006) scores were significantly lower in those with OH compared to those without.	
Francois et al. (55)	SF-8	Physical functioning: 34.3 ± 8.4 Role physical: 33.9 ± 8.6 Bodily pain: 44.0 ± 9.8 General health: 37.9 ± 6.7 Vitality: 39.3 ± 7.4 Social functioning: 39.1 ± 9.0 Role emotional: 41.1 ± 9.2 Mental Health: 43.6 ± 9.6 **Physical component summary:** 33.7 ± 8.5 **Mental component summary:** 43.1 ± 10.2		

*To avoid redundancy in our reporting, data from three longitudinal studies (26, 28, 39) have been omitted from this table; their results can be found in Table 3. We note that these three studies report follow-up data from three prior studies that are described in this table (21, 38, 56). Abbreviations: head-upright tilt test (HUT)*.

**Table 3:** Longitudinal study results.

**Study**	**Outcome measure**	**Baseline scores—population of interest (mean ±SD)**	**Follow up time point (months)**	**Follow up scores**	**Additional contextual findings**
**Vasovagal syncope**					
Baron-Esquivias et al. (26)	SF-36 (Spanish version) ^*^data reported as median and interquartile range; means and SD estimated; physical and mental component summary scores calculated based on domain scores	Physical functioning: 87 ± 22 Role physical: 56 ± 13 Bodily pain: 43 ± 12 General health: 61 ± 24 Vitality: 63 ± 34 Social functioning: 83 ± 28 Role emotional: 67 ± 75 Mental Health: 67 ± 27 **Physical Component Summary**: 50 ± 12 **Mental Component Summary**: 46 ± 18	6	Physical functioning: 87 ± 22 Role physical: 85 ± 34 **(*****p*** **=** **0.021)** Bodily pain: 43 ± 12 General health: 67 ± 30 **(*****p*** **=** **0.026)** Vitality: 58 ± 26 Social functioning: 88 ± 28 **(*****p*** **=** **0.03)** Role emotional: 67 ± 75 Mental Health: 67 ± 27 **Physical Component Summary:** 51 ± 10 **Mental Component Summary:** 46 ± 18	At follow-up time point, patients who experienced syncope recurrence during the follow up period (*n* = 33; 38.3%) had significantly worse QoL in bodily pain, general health, vitality and role emotional domains than patients who did not have syncope recurrence.
Van Dijk et al. (21)	SF-36	Physical functioning: 68 ± 28 Role physical: 46 ± 45 Bodily pain: 64 ± 28 General health: 55 ± 23 Vitality: 50 ± 26 Social functioning: 68 ± 28 Role emotional: 65 ± 43 Mental health: 67 ± 22 **Physical component summary:** 44 ± 11 **Mental component summary:** 44 ± 13	12	Physical functioning: 72 ± 29 **(*****p*** **<** **0.01**) Role physical: 61 ± 43 **(*****p*** **<** **0.01)** Bodily pain: 71 ± 29) **(*****p*** **<** **0.01)** General health: 57 ± 25) **(*****p*** **=** **0.08)** Vitality: 57 ± 26 **(*****p*** **<** **0.01)** Social functioning: 76 ± 27 **(*****p*** **<** **0.01)** Role emotional: 71 ± 40 **(*****p*** **=** **0.04)** Mental health: 72 ± 20 **(*****p*** **<** **0.01)** **Physical component summary**: 46 ± 12 **(*****p*** **<** **0.01)** **Mental component summary:** 47 ± 11 **(*****p*** **<** **0.01)**	All SF-36 scale scores improved at follow-up except general health. Effect sizes were small. Older age, higher level of comorbidity, having > 1 syncopal episode at presentation and syncope recurrence during follow up were associated with less improvement in physical component summary scores. Syncope recurrence during follow up and a neurologic or psychogenic syncope etiology were associated with less improvement in mental component summary scores.
Sheldon et al. (31) Placebo arm (n = 98 BL; *n* = 59 completed study)	SF-36	**Physical Component Summary**: 48 ± 10 **Mental Component Summary:** 46 ± 11	12	**Physical Component Summary:** 49 ± 10; NS **Mental Component Summary:** 48 ± 13; NS	
	EQ-5D-3L	EQ-5D-3L index: 0.81 ± 0.22 EQ VAS: 74 ± 18	12	EQ-5D-3L index: 0.86 ± 0.14 EQ VAS: 80 ± 14	
Ng et al. (30)	SF-36 ^*^physical and mental component summary scores calculated based on domain scores	Physical functioning: 80 ± 24 Role physical: 65 ± 40 Bodily pain: 71 ± 24 General health: 66 ± 24 Vitality: 53 ± 23 Social functioning: 76 ± 25 Role emotional: 74 ± 38 Mental health: 70 ± 19 **Physical Component Summary:** 47 ± 11 **Mental Component Summary:** 47 ± 10	12	Physical functioning: 84 ± 21 (*p* = 0.073) Role physical: 74 ± 38 (*p* = 0.066) Bodily pain: 74 ± 22 (*p* = 0.487) General health: 69 ± 21 (*p* = 0.056) Vitality: 60 ± 21 **(*****p*** **=** **0.001**) Social functioning: 83 ± 22 **(*****p*** **=** **0.004**) Role emotional: 83 ± 30 **(*****p*** **=** **0.033**) Mental health: 75 ± 16 **(*****p*** **<** **0.001**) **Physical Component Summary:** 48 ± 10 **Mental Component Summary:** 50 ± 9	Quality of life improved in patients with VVS after enrolling in a clinical trial, independent of randomization to drug or placebo and independent of syncope recurrence during follow up. Patients who experienced syncope recurrence during the follow up period tended to have lower quality of life scores at baseline (not significant) and had significantly worse quality of life scores in 5 SF-36 domains (social functioning, physical functioning, role physical, general health, bodily pain) at the 12-month follow-up time point. However, the overall improvement in quality of life from baseline to follow up was not affected by syncope recurrence during the follow up period.
Lévesque et al. (39)	QLSI	Global QoL: 6.2 ± 5.32 Health: 7.9 Cognitive: 8.4 Social: 3.0 Marital relationships: 5.1 Leisure times: 6.1 Work: 5.2 Household chores: 5.5 Affectivity: 10.7 Spirituality: 0.7	2	Global QoL: 4.0 ± 3.60 **(*****p*** **<** **0.0001)** Health: 5.5 **(*****p*** **<** **0.010)** Cognitive: 5.3 **(*****p*** **<** **0.010)** Social: 2.6 Marital relationships: 3.0 Leisure times: 3.5 **(*****p*** **<** **0.010)** Work: 3.6 *p* **<** **0.010** Household chores: 3.0 **(*****p*** **<** **0.010)** Affectivity: 6.0 **(*****p*** **<** **0.010)** Spirituality: 1.4 **(*****p*** **<** **0.0001)**	After controlling for lifetime syncope episodes, a significant improvement in global QoL and health, cognitive, leisure, work, household chores and affectivity QoL subscales was observed at 2- and 6-months following HUT (baseline). Spirituality subscale scores worsened at 2 months, then improved at 6 month follow up (compared to 2-month time point). QoL 2 months following HUT was worse in those who experienced a greater number of lifetime syncope episodes, those with little reduction in the syncope/presyncope frequency during follow up, those with anxiety/depressive disorders at baseline and a worse psychological profile at baseline. Younger age, reduced frequency of syncope/presyncope, a better baseline psychological profile at baseline (Psychiatric Symptom Index and Self-efficacy) and improvements in psychological profile during follow up were all associated with improvement in QoL during follow up. There was no effect of sex on quality of life improvement in the follow up period. There was a significant interaction between sex and syncope type; men with VVS (positive tilt test) exhibited better QoL compared to men with unexplained syncope (negative tilt test) (*p < 0.017*). All patients diagnosed with VVS reported receiving education and treatment for syncope, compared to only 53% of patients with unexplained syncope. Treatment and education for syncope were related to improvements in self-efficacy (*p < 0.016*), which was determined to be a significant predictor of improvements in quality of life (*p = 0.002*).
			6	Global QoL: 3.7 ± 3.74 **(*****p*** **<** **0.0001)** Health: 5.3 **(*****p*** **<** **0.010)** Cognitive: 4.9 **(*****p*** **<** **0.010)** Social: 2.4 Marital relationships: 4.2 Leisure times: 3.5 **(*****p*** **<** **0.010)** Work: 3.0 **(*****p*** **<** **0.010)** Household chores: 2.4 **(*****p*** **<** **0.010)** Affectivity: 5.7 **(*****p*** **<** **0.010)** Spirituality: 0.9 **(*****p*** **<** **0.0001 vs. 2 month)**	
Gibbons et al. (48) POTS (control group *n* = 29)	EQ-5D-3L	EQ VAS: 64 ± 9	6	EQ VAS: 66 ± 8 **(*****p*** **=** **0.52)**	No significant difference in EQ VAS scores at 6-month follow-up in the control group.

*Bold text denotes statistical significance*.

### Evidence on the Impacts of Syncopal Disorders on Overall Quality of Life

Compared with control or reference populations, SF-36 and RAND-36 physical and mental health summary scores were significantly reduced in patients with VVS (10, 28). In patients with POTS, one study using the SF-36 showed that both physical and mental health summary scores were reduced compared to healthy controls (46), while two others found only the physical component summary score to be impaired (41, 43). In a study using the RAND-36, both physical and mental health composite scores were impaired in patients with POTS, however the deficits in the physical health composite score were much larger than in the mental health composite score (49). No studies reported a greater impact on physical as opposed to mental health components for patients with VVS and OH, and in fact one study reported that in VVS the psychosocial impairment was greater than the physical impairment (7).

VAS (EQ VAS or global health) scores were significantly reduced in VVS, POTS, and OH patient populations (10, 49, 54), and EQ-5D-3L index scores were additionally found to be reduced in patients with OH (54). Two studies showed that total PedsQL scores (child self-reported) were significantly reduced in children with VVS compared to healthy controls (8, 35).

### Evidence on the Impacts of Syncopal Disorders on Quality of Life Domains

Three studies showed that all domain scores of the SF-36 and RAND-36 instruments were impaired in patients with VVS (10, 28, 29). In patients with POTS, one study showed that all SF-36 domain scores were reduced compared to healthy controls (46), while another study using the SF-36 found that quality of life was predominantly impaired in the physical health domains, noting impairments in physical functioning, role physical, bodily pain, general health, vitality and social functioning domains, but not role emotional or mental health domains (9). RAND-36 scores were reduced in all domains compared to controls, but the quality of life impairments were determined to be greater in physical health domains, with less severe deficits in the domains of emotional wellbeing and role limitations due to emotional problems (49).

Studies using the EQ-5D-3L showed that, in all five dimensions (mobility, usual activities, self-care, pain/discomfort and anxiety/depression) of the instrument, the prevalence of individuals reporting some or extreme problems was increased in patients with VVS, POTS, and OH compared to control or reference data (32, 49, 54). Two studies evaluated quality of life in pediatric patients with VVS using the PedsQL and reported impairments in psychosocial health and emotional functioning domains (8, 35); one detected additional impairments in school and physical functioning domains (8), and the other reported impaired social functioning (35).

### Key Factors Influencing Quality of Life Outcomes in Patients With Syncopal Disorders

#### Duration of Follow-Up Period

The evolution of quality of life in patients with VVS was evaluated after a follow-up period, without interventional treatment, in 3 studies (Table 3). Two of these studies evaluated quality of life using the SF-36. Baron-Esquivas et al. showed that after 6 months, quality of life scores improved in 3 of 8 domains (role physical, general health, and social functioning) (26), while Van Dijk et al. found that after one year, all SF-36 domain and summary scores were improved, with the exception of the general health domain (21). Lévesque et al. evaluated changes in quality of life using the QLSI and observed that significant improvements in global quality of life and quality of health, as well as cognitive, leisure, work, household chores and affectivity subscales, emerged after 2 months and were maintained after 6 months (39). Additional evaluations of longitudinal changes in quality of life in patients with VVS were available in 2 interventional studies. In the POST1 study, a clinical trial evaluating the effectiveness of metoprolol for the treatment of VVS, there was no improvement in SF-36 summary scores, the EQ-5D-3L index, or EQ VAS in the placebo arm of the study (31). Similarly, in patients with POTS, there was no improvement in EQ VAS scores in the control arm of an exercise intervention study after 6 months (48). However, when Ng et al. evaluated the impact of enrolling in a clinical trial on quality of life in patients with VVS, combining data from the POST1 and POST2 (fludrocortisone intervention) studies, they reported that, after 12 months, quality of life improves, independent of randomization to drug or placebo (30).

#### Frequency and Recurrence of Syncopal Events

Of the studies evaluating quality of life in patients with VVS, 12 explored the relationship between quality of life outcomes and the frequency or recurrence of syncopal events. Six studies showed that quality of life impairments were exacerbated in patients who experienced more frequent, or a greater number of lifetime syncopal episodes (28, 29, 32, 34, 38, 56), and 3 studies that evaluated quality of life over time showed that quality of life improvements were diminished in patients who experienced syncope recurrence during the follow up period (21, 26, 39). Another study that evaluated quality of life over time showed that, although patients who experienced syncope recurrence during the follow-up period had worse quality of life at the 12 month follow-up time point compared to those who did not, quality of life improved over the 12 month follow up period independent of whether patients experienced syncope recurrence (30). Two studies did not find quality of life scores to be significantly associated with the frequency or recurrence of syncope (36, 37).

#### Autonomic Symptom Severity and Duration

Several studies explored the relationship between autonomic symptom severity and/or duration and quality of life. In patients with VVS, the presence of presyncopal episodes was associated with decreased physical and mental component summary scores on the SF-36, and a shorter duration of symptoms was additionally associated with a reduced mental component summary score (28). In patients with POTS, several studies have shown increased orthostatic intolerance symptom severity to be correlated with reduced quality of life, predominantly in physical health domains (9, 41, 42, 45), with some evidence that mental health domains are also affected (45).

Composite autonomic symptom scores have also been shown to be correlated with quality of life in patients with POTS. Increased autonomic symptom severity (COMPASS-31) was associated with reduced PROMIS-10 physical and mental health scores, although there were no significant relationships between symptom duration (time since diagnosis, time since symptom onset) and quality of life (50). Similarly autonomic symptom severity (Autonomic Symptom Profile) was associated with reduced SF-36 physical and mental component summary scores (9). While orthostatic intolerance symptoms had the strongest correlation with the physical component summary score, most other autonomic symptom scales were also correlated (upper gastrointestinal symptoms, constipation, bladder dysfunction, pupillomotor symptoms, vasomotor symptoms, sleep dysfunction), suggesting that other autonomic symptoms interfere with quality of life in POTS patients (9). Sleep dysfunction, upper gastrointestinal symptoms and pupillomotor symptoms were all negatively correlated with mental component summary scores, but orthostatic intolerance symptoms were not (9). The detrimental impact of sleep problems/disturbances on quality of life in POTS patients was a common finding, with 3 studies reporting sleep problems/disturbances to be associated with quality of life impairments, in both physical and mental/psychosocial health domains (9, 41, 49).

Many additional commonly presenting POTS symptoms have been shown to be associated with impaired quality of life in physical health domains, including fatigue, pain, brain fog, exercise intolerance, palpitations, and purplish/bluish discoloration of hands and feet (41). However, in patients with POTS, the magnitude of the orthostatic heart rate increase does not appear to influence quality of life—the magnitude of the orthostatic heart rate increase was not correlated with orthostatic intolerance symptoms or SF-36 physical or mental component summary scores (45).

#### Activity Limitations/Functional Capacity/Disability Status

More patients with VVS and POTS report activity limitations and require help with routine needs, personal care or activities of daily living compared to healthy controls (32, 49, 51). Hutt et al. showed that a large proportion of patients with POTS have abnormally low functional capacity for their age and sex (44% of patients in their study), a finding that was more common in younger patients (44). Furthermore, patients with POTS with a low functional capacity had significantly lower SF-36 physical component summary scores compared to patients with normal functional capacity (44). In terms of employment, 24% of patients with POTS reported their employment status as disabled or unable to work because of their condition, and these patients had decreased scores in all domains of the SF-36 except mental health and reduced physical component summary scores (9).

#### Comorbid Conditions

Four studies explored the relationship between comorbidities and quality of life in patients with VVS. These studies showed that increased comorbidity (number of comorbid conditions or Charlston comorbidity index) was associated with decreased EQ VAS scores (32), and decreased physical domain scores on the SIP (7) and SF-36 (28). Moreover, in a longitudinal study, increased comorbidity was predictive of less improvement in the SF-36 physical component summary score at the 1-year follow-up time point (21).

#### Mental Health/Psychological Factors

The impact of mental health and psychological conditions on quality of life was evaluated in patients with VVS in 7 studies. Three of these studies reported the level of psychological distress (7) and prevalence of psychiatric disorders (10, 29), including anxiety and depression, to be higher in patients with VVS, while one study reported levels of depression and anxiety to be similar in patients with VVS and controls (40). All SF-36 domain scores, except bodily pain, were significantly lower in patients with psychiatric disorders than those without (29), and all subscales of the Symptom Checklist 90 (SCL-90), a measure of psychological distress, were significantly correlated with SIP psychosocial scores (7). The severity of anxiety, depression and anxiety sensitivity were all weakly but significantly correlated with RAND-36 physical and mental health composite scores, and almost all RAND-36 subscales, in patients with VVS, but not in healthy controls (10). In contrast, Broadbent et al. found that anxiety and depression were negatively correlated with quality of life variables in both patients with VVS and healthy controls, but reported that these relationships were stronger in the patient group (40). QLSI scores were poorer in patients with anxiety or depressive disorders (38) and in the longitudinal follow-up study, patients with more severe anxiety or depression and/or a worse psychological profile (Psychiatric Symptom Index) at baseline, and patients who experienced reductions in self-efficacy over the follow up period, had worse quality of life at the 2-month follow up time point (39).

In a study of pediatric patients with VVS, psychological factors (Child Behavior Checklist) were not associated with child self-reported PedsQL scores (36). Child self-reported psychosocial and total quality of life scores were, however, significantly related to indices of parent stress (Parent Stress Index: parent-child dysfunctional interaction scale, difficult child scale, total parent stress scale).

One study evaluated the relationships between mental health/psychological factors and the physical functioning domain of the SF-36 in POTS patients, finding that catastrophizing (Coping Strategies Questionnaire) and depressive (Beck Depression Inventory) symptoms were significantly related to physical functioning (42).

#### Age, Sex, and Race

Many studies investigated the impact of age and sex on quality of life in patients with VVS. In adults, some studies reported that older age was associated with decreased, or less improvement in (after follow-up), quality of life, predominantly in physical health domains (7, 21, 38, 39, 56). Despite finding that quality of life scores were reduced in older patients, syncope burden was not increased in older individuals, in fact, recurrence was more common in younger patients (39). When considering sex, SF-36 domain scores (56), PCS scores (28) and RAND-36 physical, mental and general health composite scores, as well as physical functioning, role physical, social functioning and pain domain scores (53) were increased in males with VVS. The negative relationship between SF-36 domain scores and increasing age was found to be stronger in women than in men with VVS (56). Other studies in adults with VVS did not find generic quality of life measures to be influenced by age (29, 32) and sex (21, 32, 38, 39). Two pediatric VVS studies explored these relationships and did not find any significant associations between age or sex and quality of life (8, 36). Neither study included very young children with reflex syncope who tend to present with reflex asystolic syncope (RAS), often associated with anoxic seizures.

POTS predominantly affects young women, 92% of POTS patients in the included studies in this review were female, making it difficult to evaluate the impact of sex on quality of life in POTS. One study evaluated the influence of sex on quality of life in this population, comparing quality of life between VVS and POTS patients (53). They showed that within the POTS group, males (*n* = 12) scored lower than females (*n* = 158) in the energy/fatigue domain and on the mental and general health composite scores of the RAND-36 (53). They also reported that when data from VVS and POTS patients were stratified by sex rather than diagnosis, females reported significantly lower scores in all domains of the RAND-36 except emotional wellbeing and role emotional domains. The physical health composite scores were lower in females, but there was no effect of sex on mental or general health composite scores. No studies reported analyses directly evaluating the effect of age on quality of life in POTS patients, however impairments in functional capacity, which are associated with decreased quality of life in physical domains, are more common in younger patients (44). No studies investigated the impact of age or sex on quality of life in patients with OH or CSH.

No study examined the impact of race on quality of life in patients with syncopal disorders, and in fact only 6 of the included studies provided any data on participant race (8, 9, 42, 47, 50, 55).

#### Tilt Test Outcome/Diagnosis

In patients with VVS, a few studies investigated the impact of tilt test outcome on quality of life. Rose et al. and Baron-Esquivas et al. reported that there were no significant differences in EQ VAS scores, or SF-36 domain scores, respectively, between patients with VVS with positive and negative tilt tests (32, 56). In contrast, St-Jean et al. and Lévesque et al. found that there was a significant main effect of syncope diagnosis, and a significant interaction between sex and syncope diagnosis, when analyzing quality of life scores. Men with a negative tilt test outcome (determined to have unexplained syncope) had poorer quality of life than men with a positive tilt test (determined to have VVS) (38, 39). They further demonstrated that education and treatment significantly improve self-efficacy, which is a significant predictor of quality of life, and showed that patients with unexplained syncope were less likely to receive education and treatment than those diagnosed with VVS (39). Van Dijk et al. evaluated the impact of syncope diagnosis on the evolution of quality of life outcomes after one year (21). The syncope population in their study (*n* = 268) was principally patients with VVS (77%; *n* = 205), but also comprised patients with cardiac (8.2%; *n* = 22), neurological (3.0%; *n* = 8), and psychogenic etiologies (4.9%; *n* = 13), as well as patients without a formal diagnosis (7.5%; *n* = 20), and they observed that SF-36 mental component summary scores improved less in patients with a neurological or psychological syncope diagnosis over the follow up period. Another study evaluated the impact of the subtype of VVS (VASIS classification) on EQ-5D-3L index and EQ VAS scores, and found that quality of life did not differ between VASIS subtypes (34).

### Meta-Analyses of Overall and Domain Quality of Life Scores: Comparisons to Normative Data and Across Syncopal Disorders

**Figure 2:**
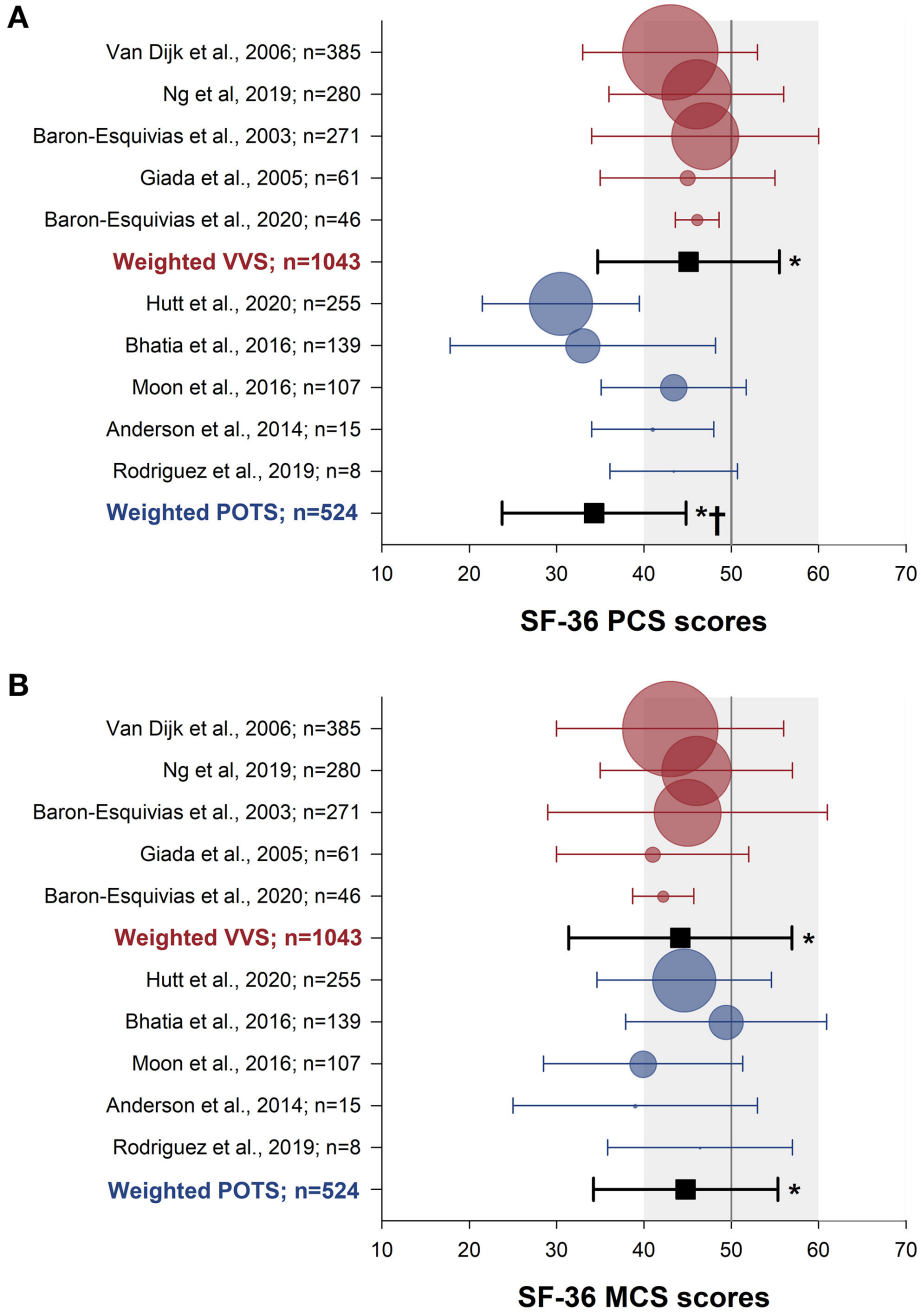
Meta-analysis of SF-36 summary scores in patients with VVS and POTS compared to normative data. Physical **(A)** and mental **(B)** component summary scores are shown for patients with VVS (27–30, 56) (red) and POTS (41, 43–46) (blue). Data for each study are presented as the mean (circle) and standard deviation (whiskers) with the size of the circle proportional to the study sample size. Weighted means and standard deviations for combined data for patients with VVS and POTS are indicated with black squares. Vertical lines and gray shading denote the mean and standard deviation based on US normative data (*n* = 2,474) (58). *Significantly different to US normative data at the 0.001 level of significance.^†^Significantly different to the VVS group, at the 0.001 level of significance.

**Figure 3:**
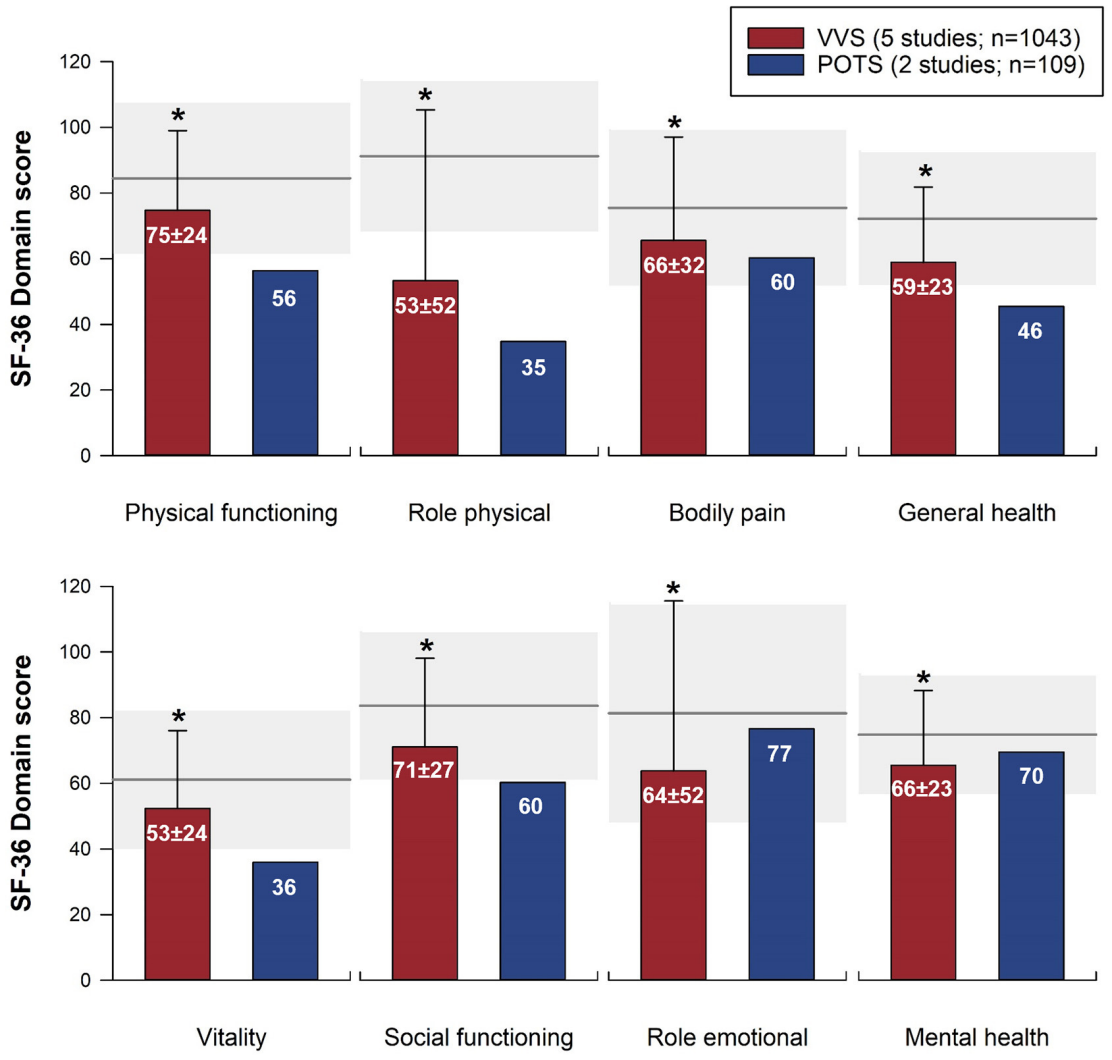
Meta-analysis of SF-36 domain scores in patients with VVS and POTS compared to normative data. SF-36 domain scores are shown for patients with VVS (26–30) and POTS (9, 46). Bars represent weighted means and standard deviations for patients with VVS and POTS. Note that the two POTS studies did not report a measure of error; the mean scores from these studies are presented for reference, but have not been included in the statistical analysis. Horizontal line and gray shading denote the mean and standard deviation based on US normative data (*n* = 2,474) (58). *Significantly different to US normative data at the 0.001 level of significance.

The SF-36 (version 1) was the most frequently used instrument, used in 15 studies. Physical and mental component summary scores for the SF-36 were available in 10 studies with distinct patient samples (5 VVS; 5 POTS). SF-36 physical and mental component summary score data from individual studies, and aggregate means and standard deviations are shown in Figure 2. Patients with VVS (*n* = 939; physical component summary score: 45.1 ± 10.4; mental component summary score: 44.2 ± 12.8) and POTS (*n* = 524; physical component summary score: 34.3 ± 10.5; mental component summary score: 44.8 ± 10.6) exhibited significant reductions in physical and mental component summary scores relative to US normative data (58) (*n* = 2,474; physical component summary score: 50.0 ± 10.0; mental component summary score: 50.0 ± 10.0; *p* < 0.001). Physical component summary scores were significantly lower in patients with POTS compared with those with VVS (*p* ≤ 0.001), while mental component summary scores were similar between VVS and POTS groups (*p* = 0.295). SF-36 domain scores, shown in Figure 3, were reported in 7 studies (5 VVS; 2 POTS). Unfortunately, no measure of error was reported in either of the POTS studies. Although we were unable to include these data in our statistical analyses, we have calculated aggregate means for the POTS population and shown them in the figure for interest. Scores in all 8 SF-36 domains were significantly reduced in patients with VVS compared to US normative data (*p* < 0.001).

Proportions of participants reporting limitations or problems across the EQ-5D-3L domains were presented in one VVS (32), one POTS (49) and one OH study (54), these data are compiled in Table 4. Significantly more patients with VVS reported limitations compared to US and regional Canadian (Alberta) normative data (59) in four of five EQ-5D-3L domains—mobility (*p* ≤ 0.043), usual activities (*p* ≤ 0.001), self-care (*p* ≤ 0.004) and anxiety/depression (*p* ≤ 0.001). More patients with OH reported limitations or problems in all domains of the EQ-5D-3L compared to US (*p* ≤ 0.005) and Canadian (*p* ≤ 0.006) norms, and more OH patients reported limitations or problems compared to patients with VVS in the mobility (*p* < 0.001) and pain/discomfort (*p* = 0.014) domains. More POTS patients reported limitations in all five domains of the EQ-5D-3L compared to US and regional Canadian norms (*p* < 0.001). Furthermore, more POTS patients reported limitations or problems in the usual activities and self-care domains compared to patients with VVS (*p* < 0.001) or OH (*p* ≤ 0.024), and in the mobility, and pain/discomfort domains compared to patients with VVS (*p* < 0.001).

**Table 4:** Percentage of individuals reporting limitations or problems (i.e., a level 2 or level 3 response) in EQ-5D-3L domains in patients with VVS, POTS, and OH compared to population/regional norms.

	**Mobility**	**Self-care**	**Usual activities**	**Pain/discomfort**	**Anxiety/depression**
VVS (*n* = 136) (32)	25.9^*^	9.0^*^	37.2^*^	49.2	43.4^*^
POTS (*n* = 44) (49)	60^*^^†^	35^*^^†^^‡^	95^*^^†^^‡^	81^*^^†^	56^*^
OH (*n* = 117) (54)	53.8^*^^†^	16.2^*^	28.2^*^	67.5^*^^†^	45.3^*^
Population normative data					
*United States (n = 38,678)* (59)	18.5	3.7	17.9	48.3	23.2

* *Significantly different when compared with normative data from the United States, at the 0.05 level of significance*.

† *Significantly different to the VVS group, at the 0.05 level of significance*.

‡ *Significantly different to the OH group, at the 0.05 level of significance*.

**Figure 4:**
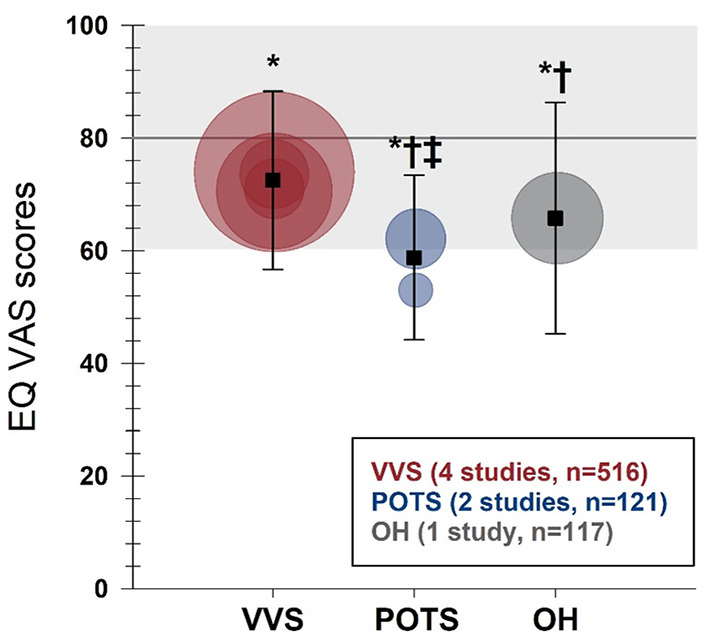
Meta-analysis of EQ VAS in patients with VVS, POTS, and OH compared to United States normative data. EQ VAS scores are shown for patients with VVS (30, 31, 33, 34), POTS (48, 49) and OH (54). Circles represent mean scores from individual studies, with the size of the circle proportional to the study sample size. Weighted means and standard deviations for combined data for patients with VVS, POTS, and OH are indicated with black squares. Horizontal line and gray shading denote the mean and standard deviation based on US normative data (*n* = 38,678) (59). *Significantly different to US normative data at the 0.001 level of significance.^†^Significantly different to the VVS group, at the 0.001 level of significance.^‡^Significantly different to the OH group, at the 0.01 level of significance.

EQ VAS scores (including global health VAS scores reported by Ng et al.) were reported in seven studies (4 VVS; 2 POTS; 1 OH). Scores from individual studies, as well as weighted means and standard deviations for each group are shown in Figure 4. Compared to US normative data (59) (*n* = 38,678; EQ VAS score: 80.0 ± 19.7), patients with VVS (*n* = 516; EQ VAS score: 72.5 ± 15.8), POTS (*n* = 121; EQ VAS score: 58.8 ± 14.6) and OH (*n* = 117; EQ VAS score: 65.8 ± 20.5) all reported significant reductions in their perceived overall health (*p* < 0.001). EQ VAS scores were significantly lower in patients with POTS and OH compared to patients with VVS (*p* ≤ 0.001) and furthermore, patients with POTS reported lower scores relative to patients with OH (*p* = 0.006).

The PedsQL was used to quantify quality of life in pediatric patients with VVS in 3 studies. Aggregate physical health, psychosocial health and total PedsQL scores were all significantly reduced (*p* < 0.001) in the VVS group (*n* = 287; physical health: 75.2 ± 16.4; psychosocial health: 72.8 ± 17.2; total score: 73.6 ± 14.2) compared to US normative data (60) [physical health (*n* = 959): 80.2 ± 19.3; psychosocial health (*n* = 958): 79.4 ± 15.7; total score (*n* = 960): 79.6 ± 15.3]. Subdomain scores within the psychosocial health dimension were available in 2 VVS studies. Emotional functioning [VVS (*n* = 162): 65.9 ± 20.1; US (*n* = 958): 78.10 ± 20.6] and school functioning [VVS (*n* = 162): 65.4 ± 20.2; US (*n* = 933): 75.9 ± 19.7] scores were significantly reduced (*p* < 0.001) in patients with VVS compared to US norms. Social functioning [VVS (*n* = 162): 81.6 ± 17.9; US (*n* = 958): 84.1 ± 18.5] scores were not different between groups (*p* = 0.116).

## Discussion

This paper represents the first comprehensive review of the impact of orthostatic syncope and presyncope on quality of life, as measured by generic quality of life instruments. With our search strategy and study selection protocol, 36 peer-reviewed articles were identified, the majority of which evaluated quality of life in patients with VVS and POTS, with a paucity of research evaluating the impact of OH and CSH on quality of life. A key finding of this review is that quality of life is consistently reported to be impaired in patients with syncopal disorders. Of note, all 15 papers that statistically compared quality of life in patients with orthostatic syncope to control or reference populations, detected quality of life impairments in patients with syncopal disorders. Our meta-analyses combined available SF-36, EQ-5D-3L and PedsQL data, enabling comparisons to population normative data and between syncopal disorders. Analyses of aggregate data showed that SF-36 physical and mental component summary scores are significantly impaired in patients with VVS and POTS; total, physical, and mental health PedsQL summary scores are significantly reduced in pediatric patients with VVS; EQ VAS scores are reduced in patients with VVS, POTS and OH; and more patients with VVS, POTS, and OH report limitations or problems in all domains of the EQ-5D-3L compared to normative population data.

While both physical and mental health dimensions of quality of life are reported to be impaired in patients with POTS, several studies reported that quality of life deficits in POTS patients are more profound in physical health domains than mental health domains (9, 41, 43, 49). The results of our meta-analysis are compatible with this finding. Furthermore, patients with POTS who are disabled and unable to work because of their condition report decreased scores in all SF-36 domains except mental health, and report reduced physical, but not mental, component summary scores relative to patients who are able to maintain school/work responsibilities (9). Together, this evidence suggests that quality of life deficits in POTS are largely due to physiological factors and resulting physical impairments, rather than psychological problems. While one study in our review found catastrophizing and depressive symptoms in patients with POTS to be significantly related to the physical functioning SF-36 domain score (42), the lifetime prevalence of major depression and anxiety disorders has been previously shown to be similar between patients with POTS and the general population (61). This relationship may reflect the psychological toll of the high physical symptom burden in patients with POTS, with impairments affecting multiple systems (cardiovascular, gastrointestinal, urinary, vasomotor, sleep, etc.) that contribute to the disabling nature of this disorder (9, 41, 49), and this is supported by the observation that quality of life was more severely impaired in those with more severe autonomic symptoms affecting multiple domains. Accordingly, improving the management of physical symptoms is a key priority for patients with POTS to increase quality of life in physical health domains, with a likely benefit of indirect improvements in mental health related quality of life.

A greater impact on physical health, as opposed to mental health dimensions of quality of life was not reported in patients with VVS. In fact, one study reported that psychosocial quality of life impairments were greater than physical quality of life deficits in patients with VVS (7). Our meta-analysis of SF-36 summary scores showed that, in patients with VVS, overall deficits in physical and mental components were similar. However, quality of life was reported to be more impaired in patients with VVS who also had other comorbid conditions, and this is similar to the greater impairment in quality of life in patients with POTS with more severe autonomic symptoms affecting multiple systems—when orthostatic syncope is associated with other physical impairments, quality of life is more severely impacted. In contrast to patients with POTS, several studies have reported the prevalence of psychiatric disorders and severity of psychological distress to be increased in patients with VVS (7, 10, 29, 38). Moreover, the presence and severity of psychiatric disorders are associated with reduced quality of life in this population, more so than in healthy controls, and are associated with less improvement in quality of life after follow-up (10, 29, 39, 40). The frequency of syncopal events is a major factor influencing quality of life outcomes in patients with VVS. Increased event frequency, or syncopal recurrence during follow up, are associated with reduced quality of life in both physical and mental health domains in patients with VVS. Syncopal recurrence is also more common in patients with VVS who have concurrent psychiatric disorders (29), and conversely, patients who report more frequent syncopal events, are more likely to have a concurrent psychiatric disorder or psychiatric symptoms (62, 63). Whether psychological distress predisposes to syncope in susceptible individuals, or whether syncope contributes to psychological distress, is not clear. Perhaps it is more likely that both are true, resulting in a positive feedback loop. Certainly, this evidence suggests that, in addition to standard management practices, addressing mental health concerns in patients with VVS is a priority, especially in patients experiencing frequent events.

Our findings highlighted the severe impact of syncopal disorders on quality of life using established clinical tools. The negative impact on quality of life also resonated with our stakeholder communities, community partners and patient advocacy groups, who highlighted that recurrent orthostatic syncope leads to stress, fear, anxiety, distress and psychological disorders, impairing the ability of affected individuals to fully participate in activities and lead active independent lives (64). Given that orthostatic syncope is not life-threatening, stake-holder communities perceive that the impact on quality of life is under recognized, with symptoms frequently ignored or dismissed as trivial (64). Many individuals also experience injury associated with the syncopal event. We note that no studies evaluated the impact of injuries sustained due to syncope, or the impact of injury treatment and recovery (hospital stays, missed school/work, severity of injury) on quality of life.

One study in our review directly compared quality of life between patients with VVS and POTS, finding that RAND-36 domain scores were significantly reduced in patients with POTS in physical functioning, role physical, energy and fatigue, social functioning, pain and general health domains, while role emotional scores tended to be lower in patients with VVS (*p* = 0.052), although this did not achieve statistical significance. Impairments in overall physical, and general health composite scores were greater in POTS patients, while the degree of impairment in the mental health component was greater in patients with VVS (53). Our meta-analysis similarly showed that SF-36 physical component summary scores are significantly reduced in patients with POTS, compared to patients with VVS, although mental component summary scores were similar between groups. Overall, quality of life in patients with syncopal disorders, quantified by the SF-36 or RAND-36 (53), appears to be most impaired in the role physical domain, reflecting that patients with VVS and POTS experience limitations in their ability to complete work or usual activities because of physical problems. Our meta-analysis of EQ-5D-3L domains further showed that compared to patients with VVS, patients with POTS report significantly more limitations or problems in mobility, usual activities, self-care and pain/discomfort domains, but report similar scores in the anxiety/depression domain.

No studies investigated quality of life in patients with CSH and research investigating quality of life outcomes in patients with OH is scarce; only two studies in this review evaluated quality of life in this group. Both studies identified significant impairments in quality of life in patients with OH, particularly in domains associated with physical quality of life (54, 65). Of note, OH and CSH are disorders that predominantly affect older adults, suggesting that research examining quality of life in older adults with syncopal disorders is relatively lacking, and should be prioritized, particularly given that OH and CSH are associated with significant morbidity and mortality in older adults. Furthermore, young children presenting with reflex syncope are often diagnosed with RAS. We note that no studies investigated the impact of RAS on quality of life. RAS attacks in young children cause fear and anxiety for both the patient and the caregiver (s), and future studies should aim to address this key knowledge gap. While our review was focused on the impact of *orthostatic* syncope and pre-syncope on quality of life, we recognize that other etiologies of syncope (e.g., cardiac syncope, psychogenic pseudosyncope) also have a negative impact on quality of life (21, 66).

While comparing quality of life between patients with syncope or presyncope and other chronic health conditions was not an aim of this study, these comparisons were reported in five studies. In patients with VVS, SF-36 scores were similar to patients with heart failure in 5 of 8 domains (56), WHO-QOL-BREF impairments were comparable to patients with epilepsy (37), reductions in health satisfaction were similar to patients with coronary artery disease (40), and total PedsQL scores were comparable to patients with asthma, end-stage renal disease, obesity and structural heart disease, and further reduced compared to patients with diabetes mellitus (8). Quality of life impairments in patients with POTS, as quantified by the SF-36, were similar to patients with congestive heart failure or chronic obstructive pulmonary disease (9). Two additional studies placed VVS and POTS quality of life scores in context with scores from other patient populations, but did not perform statistical comparisons. One of these studies stated that the degree of quality of life impairment in patients with VVS, evaluated using the SIP, was similar to patients with rheumatoid arthritis and chronic low back pain (7), the other reported that RAND-36 scores in patients with POTS were comparable to patients with rheumatoid arthritis and end-stage renal disease (49).

The gold standard for orthostatic stress testing is a head-up tilt test with combined, graded, lower body negative pressure, however, clinical tilt protocols vary in duration and may or may not involve pharmacological provocation to ensure a defined end point is reached (67). These clinical tilt protocols have generally poor sensitivity and specificity which has obvious impacts on diagnostic confidence and therefore on treatment options and quality of life. While two studies in our review found that tilt test outcome did not significantly affect quality of life (32, 56), St-Jean et al. and Lévesque et al. found that men with a negative tilt test outcome (determined to have unexplained syncope) had poorer quality of life than men with a positive tilt test (determined to have VVS). Furthermore, 100% of patients with a diagnosis of VVS reported receiving education/treatment for their condition, compared to 53% of patients with unexplained syncope (38, 39), reflecting that patients are often not given education and resources when the diagnosis is in question. Interestingly, quality of life was found to improve throughout the follow-up period, regardless of tilt outcome, perhaps reflecting the importance of the physician interaction, and patient reassurance (39). Of note, there was a disconnect between patients and their treating physicians in terms of patient education; of the patients for whom physicians reported having provided education and/or treatment, only 17.8% said that they had received education for their syncope, while 73.3% reported receiving treatment. This suggests that there is a need to improve physician-patient communication regarding syncope education and management recommendations. Patient education and treatment, including lifestyle advice, facilitate self-efficacy, which is a significant predictor of quality of life (39), thus clear communication of this information is key.

Cardiac syncope is not an autonomic disorder, and so we excluded quality of life studies focused on cardiac syncope patients in this review. The two articles reporting the results of the Dutch Fainting Assessment Study (21, 28) included patients with a diverse range of syncopal etiologies, but as these studies principally comprised patients with VVS (75 and 77%), we characterized the study population as VVS and included the data. We do not believe the inclusion of the cardiac patients to have significantly influenced the quality of life scores. As previously discussed, there is some evidence that quality of life scores are similar between patients with VVS and patients with coronary artery disease, structural heart disease and heart failure.

For the purposes of this review, we were not interested in and did not evaluate the impact of interventions on quality of life in patients with orthostatic syncope and presyncope. However, it is important to note that six (3 VVS, 2 POTS, 1 OH) of the included papers were interventional studies that included a quality of life assessment. Studies conducted in patients with POTS often use the change in orthostatic heart rate increases as a primary outcome measure to assess the effectiveness of interventions. One study included in this review reported that the magnitude of the orthostatic heart rate increase was not correlated with orthostatic intolerance symptoms or SF-36 physical or mental component summary scores (45). Similarly, in patients with VVS and OH, studies often use changes in orthostatic tolerance and orthostatic cardiovascular vitals, or syncopal recurrence as outcome measures, which may not be reflective of symptom burden and quality of life. Measures of symptom burden and quality of life constitute valuable additions to any study evaluating novel therapies or management options for patients with syncopal disorders, and should be employed in more interventional studies going forward.

We opted to include only generic quality of life instruments in our review, excluding disease-specific instruments. Our study aimed to determine to what extent syncopal disorders impact quality of life, and to evaluate this, we evaluated quality of life scores from patients with orthostatic syncope in context with scores from healthy controls; disease-specific tools do not lend themselves to these types of assessments.

One limitation of this review is the possibility that some relevant articles were not identified with our search strategy. The absence of formal standards for indexing quality of life and quality of life instruments, as well as the inconsistencies in the reporting of these outcome measures across studies may have influenced our ability to identify relevant articles. To minimize this possibility, we used broad search criteria and conducted our search in five academic databases. A second limitation relates to the variability in the reporting of quality of life data across studies. Many articles reported only quality of life summary scores and did not report the domain scores of their chosen quality of life instrument. We extracted all relevant data from manuscripts as they were reported and did not contact authors for supplemental information. We do not believe that this limitation diminishes the value of our findings.

One caveat to the present study was the limited ability to compare quality of life in orthostatic syncope patients based on age, sex or race because these data were seldom reported and there were few studies on syncopal disorders in older adults. Another consideration is that in this systematic review, we elected not to perform an evaluation of the quality of evidence from included studies, and we do not believe that this evaluation should be performed, given our research question and methodology. Most instruments designed to evaluate the quality of evidence in systematic reviews (e.g., GRADE, Cochrane RoB 2) are intended for systematic reviews that evaluate the impact of an intervention on clinical outcome measures, or for diagnostic or prognostic tools. These tools are not suitable for the present study, which aimed to consolidate and report evidence on the impact of orthostatic syncope and presyncope on quality of life, because study design is not likely to have influenced the quality of the data extracted. For example, in our context, baseline quality of life data extracted from a double-blind randomized control trial can not and should not be assessed as higher quality than quality of life data extracted from a cross-sectional study. We did not formally evaluate heterogeneity in the literature synthesized in the meta-analyses because in every case quality of life was found to be impaired in patients with orthostatic syncope, with remarkable homogeneity. Any differences in the effect sizes between different studies and/or sub-groups are reported in the figures and tables. Certainly, there are variables that may have influenced quality of life outcomes across studies, such as sample size, sample heterogeneity, and duration and severity of illness in recruited patients. These variables have been transparently reported in this systematic review, and their influence on study outcomes is not likely to be captured by instruments designed to evaluate the quality of evidence in interventional studies.

## Conclusions

The impact of syncopal disorders on quality of life is not trivial, with profound impairment across physical and mental health domains compared to healthy controls. Patients with POTS and with a high frequency of episodes experience particularly severe impairments to quality of life. Despite the high prevalence of orthostatic syncope in older adults, and the strong association between orthostatic syncope and morbidity and mortality, quality of life remains poorly studied in this population. Researchers and clinicians should prioritize consideration of the impact of orthostatic syncopal disorders on quality of life.

## Data Availability Statement

The original contributions presented in the study are included in the article/Supplementary Material, further inquiries can be directed to the corresponding author/s.

## Author Contributions

BH, NH, DW, and VC conceived and designed the study. BH and NH conducted the search, screened articles for inclusion, and extracted data from included studies. BH performed data analyses and data visualization. BH, DW, and VC interpreted the data and drafted the manuscript. All authors reviewed the manuscript, provided critical edits and insight, and approved the final version for submission.

## Funding

This work was funded by a grant-in-aid from the Heart and Stroke Foundation of Canada awarded to VC (Grant Number G-18-0022174). This work was also supported by a graduate student fellowship awarded to BH from the BC SUPPORT Unit Fraser Centre.

## Conflict of Interest

The authors declare that the research was conducted in the absence of any commercial or financial relationships that could be construed as a potential conflict of interest.

## Publisher's Note

All claims expressed in this article are solely those of the authors and do not necessarily represent those of their affiliated organizations, or those of the publisher, the editors and the reviewers. Any product that may be evaluated in this article, or claim that may be made by its manufacturer, is not guaranteed or endorsed by the publisher.
