# Supplementary material for: Evaluating the Impact of Orthostatic Syncope and Presyncope on Quality of Life: A Systematic Review and Meta-Analysis
Source: Front Cardiovasc Med. 2022 Feb 10;9:834879. doi: 10.3389/fcvm.2022.834879 (PMC8866568; doi:10.3389/fcvm.2022.834879)
Supplement: Supplementary file 1 [file Data_Sheet_1.docx]

Supplementary Material

# Supplementary Tables

**Appendix A: Search Strategy**

| Database | Search Term | # of results |
| --- | --- | --- |
| Pubmed | (syncope OR presyncope OR faint* OR "vasovagal syncope" OR POTS OR "postural orthostatic tachycardia syndrome" OR "orthostatic hypotension" OR "autonomic failure" OR "carotid sinus hypersensitivity") OR "orthostatic intolerance" OR "loss of consciousness" OR "drop attack" OR syncope[MeSH Terms] OR presyncope[MeSH Terms] OR fainting[MeSH Terms] OR syncope, vasovagal[MeSH Terms] OR postural orthostatic tachycardia syndrome[MeSH Terms] OR pure autonomic failure[MeSH Terms] OR orthostatic intolerance[MeSH Terms] OR orthostatic hypotension[MeSH Terms]) OR loss of consciousness[MeSH Terms] OR drop attack[MeSH Terms])  AND ("quality of life" OR "QoL"OR "HRQoL" OR "participation" OR "morbidity" OR "activities of daily living" OR "social participation" OR "community participation" OR SF-36 OR "life satisfaction" OR "mental health" OR "wellbeing" OR PedsQL OR quality of life[MeSH Terms] OR community participation[MeSH Terms] OR social participation[MeSH Terms] OR morbidity[MeSH Terms] OR activities of daily living[MeSH Terms] OR mental health[MeSH Terms]) | 4,756 |
| Web of Science | (syncope OR presyncope OR faint* OR "vasovagal syncope" OR POTS OR "postural orthostatic tachycardia syndrome" OR "orthostatic hypotension" OR "autonomic failure" OR "carotid sinus hypersensitivity" OR "orthostatic intolerance" OR "loss of consciousness" OR "drop attack")  AND ("quality of life" OR "QoL" OR "HRQoL" OR "participation" OR "morbidity" OR "activities of daily living" OR "social participation" OR "community participation" OR SF-36 OR "life satisfaction" OR "mental health" OR "wellbeing" OR PedsQL) | 2,465 |
| CINAHL | *Same as Web of Science* | 713 |
| PsychINFO | *Same as Web of Science* | 810 |
| Embase | *Same as Web of Science* | 8,467 |
| TOTAL |  | 17,211 |

**Appendix B: Summary of Quality of Life Instruments**

| 1. Instrument 2. Instrument type 3. Frequency (# of studies) | Assessment details and domains | Scoring | Population normative data |
| --- | --- | --- | --- |
| 1. EQ-5D-3L 2. HRQoL 3. 7 | The EQ-5D-3L questionnaire consists of a descriptive system and a visual analogue scale (EQ VAS). The descriptive system classifies HRQoL across five dimensions: mobility, self-care, usual activities, anxiety/depression and pain/discomfort. The EQ VAS provides a quantitative measure of an individual’s perceived overall health, with anchors labelled as ‘The worst health you can imagine’ at zero, and ‘The best health you can imagine’ at 100[1]. Earlier versions of the EQ VAS (for the EQ-5D-3L) used the labels ‘Best imaginable health state’ and ‘Worst imaginable health state’. | Descriptive system: Each dimension has three levels (1: no problems, 2: some/moderate problems, 3: extreme problems/unable to), reflecting increasing levels of impairment. A profile score for the five dimensions (e.g., 12112) can be used to describe a health state. Value sets (i.e., a set of health state values – sometimes called ‘utility scores’ or ‘index scores’ – for all 243 (3^5^) health states defined by the EQ-5D-3L) are available for many different countries and regions.[1]  EQ VAS: The respondent rates their health on a vertical VAS from zero – “The worst health you can imagine” – to 100 – “The best health you can imagine”. | U.S. population(n=38,678)[2]  Descriptive System *(% reporting any limitations or problems)*:   - Mobility: 18.5% - Usual activities:3.7% - Self-care: 17.9% - Pain/Discomfort: 48.3% - Anxiety/Depression: 23.2%   EQ-5D-3L index: 0.823 ± 0.197  EQ VAS: 80.0 ± 19.7 |
| 1. Medical Outcomes Study 36-item Short Form Health Survey (SF-36) 2. HRQoL 3. 15 | The SF-36 is a 36-item questionnaire that measures generic health status and QoL in eight domains: physical functioning (PF), role limitations due to physical health (RP), bodily pain (BP), general health (GH), vitality (VT), social functioning (SF), role limitations due to emotional health (RE) and mental health (MH). Two summary scores are formed from the 8-domains: the physical component summary (PCS) and mental component summary (MCS)[3]. | The eight domain scores are converted to 0-100 scales where higher scores indicate better functioning or well-being[3]. To form the PCS and MCS scales, the eight subdomain scores are aggregated using norm-based scoring methods (T-score transformation) where the scores are normalized to the U.S. population mean and standard deviation of 50 ± 10[4]. | U.S. population (n=2,474)[4]:  PF: 84.5 ± 22.9  RP: 81.2 ± 33.8  BP 75.5 ± 23.6  GH: 72.2 ± 20.2  VT: 61.1 ± 20.9  SF: 83.6 ± 22.4  RE: 81.3 ± 33.0  MH: 74.8 ± 18.0  PCS: 50.0 ± 10  MCS: 50.0 ± 10 |
| 1. SF-36 version 2 (SF-36v2) 2. HRQoL 3. 1 | This updated version of the SF-36 evaluates HRQoL in the same eight domains and provides the same two component summary scores as version 1.0. Compared to version 1.0, version 2.0 includes simpler instructions and questionnaire items, an improved layout for questions and answers, offers greater comparability between translations and cultural adaptations and uses five-level response choices instead of dichotomous responses for the items comprising the two role functioning scales[5]. | Version 2.0 of the SF-36 uses norm-based scoring algorithms (T-score transformations with mean of 50 ± 10 [SD]) for all eight domain scales in addition to the PCS and MCS scores[5]. | U.S. population[5]:  PF: 50.0 ± 10  RP: 50.0 ± 10  BP 50.0 ± 10  GH: 50.0 ± 10  VT: 50.0 ± 10  SF: 50.0 ± 10  RE: 50.0 ± 10  MH: 50.0 ± 10  PCS: 50.0 ± 10  MCS: 50.0 ± 10 |
| 1. Medical Outcomes Study 8-item Short Form Health Survey (SF-8) 2. HRQoL 3. 1 | An abbreviated, eight-item version of the SF-36. Evaluates HRQoL in the same eight domains as the SF-36[6]. | Similar to the version 2.0 of the SF-36, norm-based scale scores for each domain are calculated and PCS and MCS scores are derived using norm-based scoring methods (T-score transformations with mean of 50 ± 10 [SD])[6]. | As for Medical Outcomes Study SF-36 Version 2.0. |
| 1. RAND 36-item Health Survey (RAND-36) 2. HRQoL 3. 3 | The RAND-36 health survey evaluates HRQoL using the same 36 items as the SF-36 and, similarly, yields two summary scores (PCS and MCS) and eight domains scale scores (physical functioning, role physical, pain, general health, emotional well-being, energy/fatigue, social functioning, role emotional). The RAND-36 uses a scoring algorithm that is different to the SF-36.[7] | The eight domain scores are converted to 0-100 scales where higher scores indicate better functioning or well-being; the scoring of the BP and GH domains differ slightly between the SF-36 and RAND-36; BP scores tend to be slightly higher on the RAND-36, but GH scores tend to be similar. PCS and MCS scores are calculated using a similar method to the SF-36, with the exception that there is no assumption in the model that physical and mental health constructs are uncorrelated.[8] | U.S. population (n=2471)[7]:  Physical functioning: 70.61 ± 27.42  Role physical: 52.97 ± 40.78  Pain: 70.77 ± 25.46  General health: 56.99 ± 21.11  Emotional well-being: 70.38 ± 21.97  Energy/fatigue: 52.15 ± 22.39  Social functioning: 78.77 ± 25.43  Role emotional: 65.78 ± 40.71  Physical health composite: 50 ± 10  Mental health composite: 50 ± 10 |
| 1. Pediatric Quality of Life inventory (PedsQL) version 4.0 2. HRQoL 3. 3 | The PedsQL Generic Core Scales are 23 item questionnaires designed to evaluate the core health dimensions as delineated by the World Health Organization (physical functioning, emotional functioning, social functioning) and role (school) functioning in children and young adults. The PedsQL comprises both child self-report (Ages 5-7, 8-12, 13-18, 18-24 years) and parent proxy-report forms (Ages 2-4, 5-7, 8-12, 13-18, 18-24 years). Question phrasing is adapted for different age groups to ensure questions are developmentally appropriate. Scores are provided for each of the four core scales and three summary scores (the psychosocial health summary score, the physical health summary score and total scale score)[9]. | Each item is answered on a Likert scale from 0-4. For scoring, each item is reverse scored on a 0-100 scale and the mean of items comprising each of the four dimensions produce the scale scores. The psychosocial health summary is computed as the mean score of the items comprising the emotional, social and school functioning scales. The physical health summary score is the same as the physical functioning score. The total scale score is the mean score of all 23 items[10]. | U.S population[10]  *Child self report scores (n=933-960; age 5-18 years):*  Total score: 79.62 ± 15.26  Physical health: 80.19 ± 19.30  Psychosocial health: 79.37 ± 15.70  Emotional functioning: 78.10 ± 20.66  Social functioning: 84.09 ± 18.50  School functioning: 75.87 ± 19.71  *Proxy report scores (n=1417-1622; age 2-18 years):*  Total score: 80.87± 16.73  Physical health: 81.38 ± 23.18  Psychosocial health: 80.58 ± 16.52  Emotional functioning: 77.95 ± 20.67  Social functioning: 85.38 ± 19.17  School functioning: 77.80 ± 22.00 |
| 1. World Health Organization Brief Quality of Life Questionnaire (WHOQOL-BREF) 2. HRQoL 3. 2 | An abbreviated 26 item version of WHOQOL-100. Produces scores in 4 domains of QoL: Physical Health, Psychological, Social Relationships, and Environment, and separate scores evaluating overall QoL and general health[11]. | Each question is answered on a Likert scale of 1-5. To calculate domain scores, the mean of the items making up each domain is multiplied by 4 (to make scores comparable to the WHOQOL-100), then transformed to a 0-100 scale, where 0 is worst possible, and 100 is best possible QoL in that domain. Two questions are analyzed separately: "How would you rate your quality of life?" and "How satisfied are you with your health?”.[11] | North American population normative data not available. |
| 1. Quality of Life Systemic Inventory (QLSI) 2. QoL 3. 2 | The QLSI assesses the individual’s capacity to achieve their goals in 28 domains of their life (e.g. family life, work, leisure). These domains are classified into 9 sub-scales: health, cognitions, social, marital relationships, leisure times, work, household chores, affectivity, spirituality[12]. | Scores are provided for each of the nine sub-scales which are then used to calculate a global QoL score. Scores correspond to the gap between goals the individual has set for themselves, and their actual situation; increased scores reflect a larger gap and thus poorer quality of life[12, 13]. | Canadian population[12]  Global score = 3.8 ± 3.9  Subscales range from .85 ± 3.9 for the spirituality domain to 5.22 ± 7 for the leisure times domain. |
| 1. Healthy Days Core Module (CDC HRQOL-14) 2. HRQoL 3. 1 | The CDC HRQOL-14 is a 14-item questionnaire that is divided into three modules where HRQoL is assessed based on the number of days affected in the past month. The Healthy days Core Module asks about general, physical and mental health, and the number of days the respondent experienced poor physical or mental health. The Activity Limitations Module evaluates health related limitations, including the type of health problem, duration of limitation and need for assistance in daily activities. The Healthy Days Symptoms Module assesses symptoms, their frequency and impact on daily activities[14, 15]. | The CDC HRQOL-14 does not use summary scales or scores, individual items were designed to be individual global indicators of HRQoL and activity limitations. The only calculated measure is the “unhealthy days” index which is calculated by adding unhealthy days due to mental health and physical health, with a maximum of 30 days[14, 15]. | U.S. population (n=198,508)[16]  Mean number of days in past 30 days:  Healthy days: 24.7  Unhealthy days: 6.0±0.1  Poor physical health: 3.5±0.1  Poor mental health: 3.4±0.1  Activity limitations: 2.0±0.1  Pain: 2.6  Sad, blue, depressed:3.0  Anxious: 5.2  Sleepless: 7.6  Full of energy: 19.0  *No measure of error reported for mean number or days with pain, sad/blue/depressed, anxious, or sleepless. |
| 1. Personal Well-being Index – Adult form (PWI-A) 2. QoL 3. 1 | A seven-item questionnaire where respondents are asked to rate their satisfaction in seven domains of well-being: standard of living, health, achieving in life, personal relationships, safety, community connectedness and future security. There are two additional optional items, one that assesses general life satisfaction and one that assesses satisfaction in the domain of spirituality and religion[17]. | Each item is rated on an 11-point End-Defined Response Scale (0-10): 0 represents complete dissatisfaction and 10 represents complete satisfaction. Items are linearly converted to a 0-100 scale. Each domain can be analyzed as a separate variable, or the domain scores can be summed to yield an average score with represents “Subjective Wellbeing”. The spirituality or religion item can be included in this index, but the general life satisfaction item should be analyzed as a separate variable[17]. | North American population normative data not available. |
| 1. Sickness Impact Profile (SIP) 2. HRQoL 3. 1 | A 136-item questionnaire evaluating the impact of sickness on 12 domains of activities of daily living (ambulation, mobility, body care and movement, social interaction, communication, alertness behaviour, emotional behaviour, sleep and rest, eating, work, home management, and recreation and pass-times). The respondent is asked to check only the statements that describe them on a given day and are related to their health.[18] | The SIP yields a total score (overall measure of function), two-dimension scores (physical and psychosocial dimensions of function) and 12 category scores. Each of these scores is calculated as a percentage of the # of items checked by the respondent in the given category or dimension over the total number of items in the category or dimension. [18] | U.S. population (n=696) [18]  Total score: 3.6 ± 5.3  No normative data otherwise available[19]. |
| 1. Patient-Reported Outcomes Measurement Information System, short form (PROMIS-10 global) 2. HRQoL 3. 1 | A 10-item questionnaire that evaluates global domains of health and functioning. (Overall health, quality of life, physical health, mental health, social activities and relationships, everyday physical activities, pain, fatigue, usual social activities and roles, emotional problems)[20]. | Nine items are rated on five-point scales, while pain is rated from 0 to 10. The global health physical (physical health, physical function, pain, fatigue) and global health mental scores (quality of life, mental health, emotional problems and satisfaction with discretionary social activities) summary scores are each calculated as the sum of four PROMIS-10 items[20]. Raw summed scores undergo norm-based T-score transformations to normalize scores to a U.S. population mean and standard deviation of 50 ± 10[21]. | U.S. population[21] Global health physical: 50 ± 10  Global health mental: 50 ± 10 |

Abbreviations: Quality of life (QoL), health-related quality of life (HRQoL)

# References

1. EuroQol Research Foundation (2018) EQ-5D-3L User Guide

2. Szende A, Janssen B, Cabasés J (2014) Self-reported population health: An international perspective based on EQ-5D. Springer Netherlands

3. Ware JE, Snow KK, Kosinski M, Gandek B (1993) SF-36 Health Survey : Manual and interpretation guide. The Health Institute, New England Medical Center, Boston, Massachusetts

4. Ware JE, Kosinski M, Keller SD (1994) SF-36 Physical and Mental Health Summary Scales: a User’s Manual. Health Assessment Lab, New England Medical Center, Boston, Massachusetts

5. Ware JE (2000) SF-36 Health Survey update. Spine (Phila. Pa. 1976). 25:3130–3139

6. Ware JE, Kosinski M, Dewey JE, Gandek B (2001) How to Score and Interpret Single-item Health Status Measures: A Manual for Users of the SF-8 Health Survey. QualityMetric, Inc, Lincoln, RI

7. Hays RD, Sherbourne CD, Mazel RM (1993) The RAND 36‐Item Health Survey 1.0. Health Econ 2:217–227. https://doi.org/10.1002/hec.4730020305

8. Hays RD, Morales LS (2001) The RAND-36 measure of health-related quality of life. In: Annals of Medicine. Royal Society of Medicine Press Ltd, pp 350–357

9. Varni JW (2021) PedsQL TM (Pediatric Quality of Life Inventory TM). https://www.pedsql.org/about_pedsql.html. Accessed 24 Jun 2021

10. Varni JW, Seid M, Kurtin PS (2001) PedsQL^TM^ 4.0: Reliability and Validity of the Pediatric Quality of Life Inventory^TM^ Version 4.0 Generic Core Scales in Healthy and Patient Populations. Med Care 39:800–812. https://doi.org/10.1097/00005650-200108000-00006

11. WHOQOL group (1996) WHOQOL-BREF : introduction, administration, scoring and generic version of the assessment : field trial version. Geneva, Switzerland

12. St-Jean K, Kus T, Dupuis G, et al (2008) Quality of life in patients with recurrent vasovagal or unexplained syncope: Influence of sex, syncope type and illness representations. Appl Res Qual Life 3:235–249. https://doi.org/10.1007/s11482-009-9058-x

13. Duquette R, Dupuis G, Perrault J (1994) A new approach for quality of life assessment in cardiac patients: rationale and validation of the Quality of Life Systemic Inventory - PubMed. Can J Cardiol 10:106–112

14. Centers for Disease Control and Prevention (2000) Measuring Healthy Days. Atlanta, Georgia

15. Center for Disease Control Healthy Days Core Module: HRQOL-14 Measure . https://www.cdc.gov/hrqol/hrqol14_measure.htm. Accessed 1 Jul 2021

16. Zahran HS, Kobau R, Moriarty DG, et al Health-Related Quality of Life Surveillance --- United States, 1993--2002

17. International Wellbeing Group (2013) Personal Wellbeing Index: 5th Edition. Australian Centre on Quality of Life, Deakin University, Melbourne, Australia

18. Bergner M, Bobbitt RA, Carter WB, Gilson BS (1981) The sickness impact profile: Development and final revision of a health status measure. Med Care 19:787–805. https://doi.org/10.1097/00005650-198108000-00001

19. Wu AW, Skinner EA, Pfoh E, Steinwachs DM (2014) Sickness Impact Profile (SIP). In: Michalos AC (ed) Encyclopedia of Quality of Life and Well-Being Research. Springer Netherlands, Dordrecht, pp 5959–5963

20. Hays RD, Bjorner JB, Revicki DA, et al (2009) Development of physical and mental health summary scores from the patient-reported outcomes measurement information system (PROMIS) global items. Qual Life Res 18:873–880. https://doi.org/10.1007/s11136-009-9496-9

21. HealthMeasures (2021) PROMIS Reference Populations. In: Northwest. Univ. https://www.healthmeasures.net/score-and-interpret/interpret-scores/promis/reference-populations. Accessed 1 Jul 2021
